# Norrin-induced Frizzled4 endocytosis and endo-lysosomal trafficking control retinal angiogenesis and barrier function

**DOI:** 10.1038/ncomms16050

**Published:** 2017-07-04

**Authors:** Chi Zhang, Maria B. Lai, Lavan Khandan, Lindsey A. Lee, Zhe Chen, Harald J. Junge

**Affiliations:** 1 Department of Molecular, Cellular, and Developmental Biology, University of Colorado, Boulder, Colorado 80309, USA

## Abstract

Angiogenesis and blood–brain barrier formation are required for normal central nervous system (CNS) function. Both processes are controlled by Wnt or Norrin (NDP) ligands, Frizzled (FZD) receptors, and β-catenin-dependent signalling in vascular endothelial cells. In the retina, FZD4 and the ligand NDP are critical mediators of signalling and are mutated in familial exudative vitreoretinopathy. Here, we report that NDP is a potent trigger of FZD4 ubiquitination and induces internalization of the NDP receptor complex into the endo-lysosomal compartment. Inhibition of ubiquitinated cargo transport through the multivesicular body (MVB) pathway using a dominant negative ESCRT (endosomal sorting complexes required for transport) component VPS4 EQ strongly impairs NDP/FZD4 signalling *in vitro* and recapitulates CNS angiogenesis and blood-CNS-barrier defects caused by impaired vascular β-catenin signalling in mice. These findings provide evidence for an important role of FZD4 endocytosis in NDP/FZD4 signalling and in CNS vascular biology and disease.

## Abstract (web summary)

Multiple mechanisms regulate Wnt/ß-catenin signalling. Zhang *et al*. describe a novel regulatory pathway and show that the activator of canonical Wnt signalling, Norrin, triggers endocytosis of its receptor Frizzled4 by promoting Frizzled4 ubiquitination.

Wnt/β-catenin signalling (also referred to as canonical Wnt signalling) controls major developmental processes and is implicated in several human diseases, including vascular diseases^1^. Angiogenesis and blood-CNS barrier formation enable CNS function and require ß-catenin signalling in vascular endothelial cells^2^,^3^,^4^,^5^,^6^. Consequently, impaired blood vessel development and vascular dysfunction are implicated in inherited CNS vascular diseases and neurodegenerative diseases^7^. In the retinal vasculature, canonical signalling is mediated by the ligand NDP and a receptor complex composed of FZD4, LRP5 (low-density lipoprotein receptor-related protein 5), and the tetraspanin family member TSPAN12 (refs 8, 9). The crystal structures of NDP in complex with FZD4 show how NDP interacts with FZD4 (refs 10, 11) and genetic experiments demonstrate that NDP/FZD4 signalling is mediated by β-catenin^6^. Mice with gene disruptions in *Ndp*, *Fzd4*, *Lrp5* or *Tspan12* display overlapping retinal vascular phenotypes characterized by failure of intraretinal blood vessel development and blood-retina barrier defects^9^,^12^,^13^,^14^,^15^. In humans, mutations in *NDP*, *FZD4*, *LRP5* or *TSPAN12* genes are linked to familial exudative vitreoretinopathy (FEVR), which is characterized by retinal vascular defects, vision impairment, or blindness^16^. Thus, NDP/FZD4 signalling plays a major role in retinal physiology and in human disease.

Mechanisms of canonical signalling have been elucidated in the context of Wnt-induced ß-catenin signalling. Wnt-activated FZD receptor and LRP5/6 co-receptor inhibit glycogen synthase kinase 3 beta (GSK3β), the central effector of the β-catenin destruction complex, and thus signal the stabilization of β-catenin, which in turn forms a transcriptional complex with T-cell factor/lymphoid enhancer factor (TCF/LEF)^17^. Canonical signalling is coupled to the formation of LRP6-signalosomes, plasma membrane associated aggregates containing phospho-LRP6, dishevelled (DVL), FZD, axin, and other destruction complex components^18^, which require cellular synthesis of 4,5-bisphosphates for assembly^19^. Clathrin and the endocytic adapter complex AP2, which interacts with DVL^20^, have been reported to promote signalosome formation and signalling^21^,^22^.

LRP6 endocytosis has been studied both as a mechanism of desensitization^23^,^24^ and as mediator of a component of signalling that originates from the endo-lysosomal compartment^25^,^26^,^27^,^28^. The ligand-induced internalization of receptor, co-receptor and associated destruction complex components are thought to lead to productive signalling from endosomes and/or to sequestration of GSK3β into MVBs, thus preventing GSK3β from marking cytoplasmic β-catenin for degradation^29^,^30^. This mechanism, next to the direct inhibition of GSK3ß by the activated receptor complex, may lead to enhanced or sustained signalling^31^. Consistent with a role of the endo-lysosomal compartment in canonical signalling is the finding that endosomal acidification by V-ATPase, which is physically linked to the receptor complex via the prorenin receptor, is a requirement of Wnt/β-catenin signalling^32^. GSK3β sequestration requires the function of ESCRT complexes, which mediate the movement of ubiquitinated cargo into late endosomes. However, FZD ubiquitination has not been reported to be ligand dependent^33^ and is not known to lead to GSK3β sequestration. Instead, in the absence of WNTs, FZD ubiquitination by ZNRF3 and RNF43, two homologous transmembrane E3 ubiquitin ligases that ubiquitinate FZD receptors, induces FZD clearance from the plasma membrane to diminish signalling^34^,^35^,^36^,^37^.

FZD endocytosis has been predominantly studied in the context of non-canonical signalling. FZD4 endocytosis triggered by a combination stimulus WNT5A and PKC activator PMA (phorbol 12-myristate 13-acetate) is DVL-dependent and has been linked to planar cell polarity signalling^38^. In studies of canonical signalling, reports have focused on interactions of the endocytic machinery with LRP6 or DVL, whereas little is known about ligand-induced FZD interactions with mediators of internalization.

Here, we investigate the role of endocytosis in the canonical NDP/FZD4 signalling system. We find that NDP promotes FZD4 ubiquitination and potently triggers internalization of the NDP receptor complex components FZD4, LRP5 and TSPAN12 into the endo-lysosomal compartment. Inhibition of the MVB pathway using dominant negative VPS4 EQ strongly impairs NDP/FZD4 signalling *in vitro*. Induced expression of VPS4 EQ in vascular endothelial cells in mice recapitulates previously reported angiogenesis and blood-CNS barrier defects caused by endothelial cell-specific ß-catenin deletion. We conclude that NDP-induced FZD4 endocytosis and endo-lysosomal trafficking control retinal angiogenesis and barrier function.

## Results

### NDP triggers the internalization of its receptor complex

**Figure 1:**
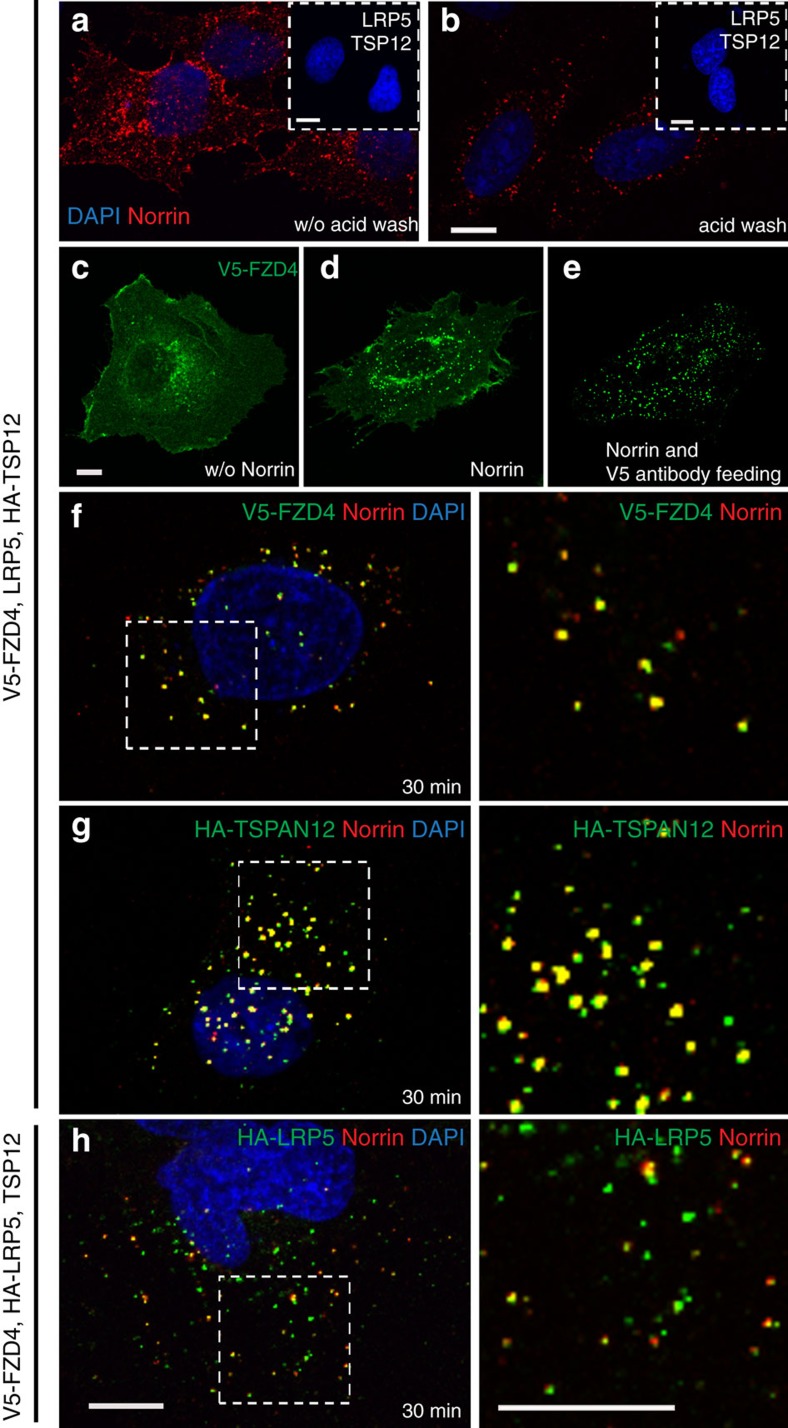
Norrin induces endocytosis of the FZD4 receptor and associated membrane proteins. (**a**) HeLa cells incubated with AP-Norrin-mycHis conditioned medium on ice were washed and then subjected to a 30 min 37 °C stimulus to induce endocytosis. Staining reveals both cell surface bound and internalized Norrin (via myc). Inset: transfection of co-receptor LRP5 and co-activator TSPAN12 is not sufficient to mediate binding or internalization of Norrin. (**b**) Removing surface bound Norrin by an acid wash step reveals specifically internalized Norrin. (**c**) FZD4 shows predominantly cell surface localization in the absence of Norrin. (**d**) Norrin-induced FZD4 internalization in a cell stained after permeabilization. (**e**) V5-FZD4 at the cell surface of intact cells was labelled with a V5 antibody. Induction of endocytosis with Norrin and subsequent acid wash allows to specifically reveal internalized FZD4 via the co-internalized V5 antibody (‘antibody feeding’). (**f**–**h**). Norrin and FZD4 co-localize in endocytic puncta. Co-activator TSPAN12 and co-receptor LRP5 are co-internalized. Boxed areas in **f**–**h** are shown enlarged on the right with the DAPI channel omitted. Internalized FZD4 is specifically revealed by V5 antibody feeding. A similar experiment without V5 antibody feeding is shown in Supplementary Fig. 1a. Scale bars, 10 μm.

To determine if NDP triggers endocytosis of the NDP-receptor complex, we transfected HeLa cells with V5-FZD4, LRP5 and HA-TSPAN12. After incubation of the cells with alkaline phosphatase (AP)-tagged NDP conditioned medium on ice, unbound NDP was removed and endocytosis enabled at 37 °C. Cells displayed both surface-bound and internalized NDP in the absence of an acid wash step before fixation (Fig. 1a), whereas washing with acidic buffer removed surface-bound NDP and revealed specifically internalized NDP (Fig. 1b; Supplementary Fig. 1a). Transfected FZD4 was essential for both cell surface binding and internalization of NDP, while LRP5 and TSPAN12 were not sufficient to mediate either process (Fig. 1a insets, see also untransfected cells in Supplementary Fig. 1a). FZD4 was detected predominantly at the cell surface in cells subjected to the internalization protocol in the absence of NDP (Fig. 1c), however, addition of NDP triggered the accumulation of FZD4 in intracellular puncta (Fig. 1d). When cells were incubated with both NDP and a V5-antibody targeting the extracellular tag of V5-FZD4, internalized antibody revealed specifically intracellular FZD4 after acid wash (Fig. 1e). Co-transfection efficiency in HeLa cells was very high (Supplementary Fig. 1b). When all receptor complex components were co-transfected, internalized NDP co-localized with each component of the receptor complex, that is, FZD4, TSPAN12 and LRP5, indicating that the entire receptor complex was endocytosed (Fig. 1f–h). We used the previously described FZD4 M157V mutation^39^ in the binding interface for NDP^10^,^11^ to impair NDP binding. When the NDP/FZD4 interaction was disturbed using the FZD4 M157V mutation, NDP-induced endocytosis was clearly reduced (Supplementary Fig. 1c–g).

### Internalized NDP and FZD4 in the endo-lysosomal compartment

**Figure 2:**
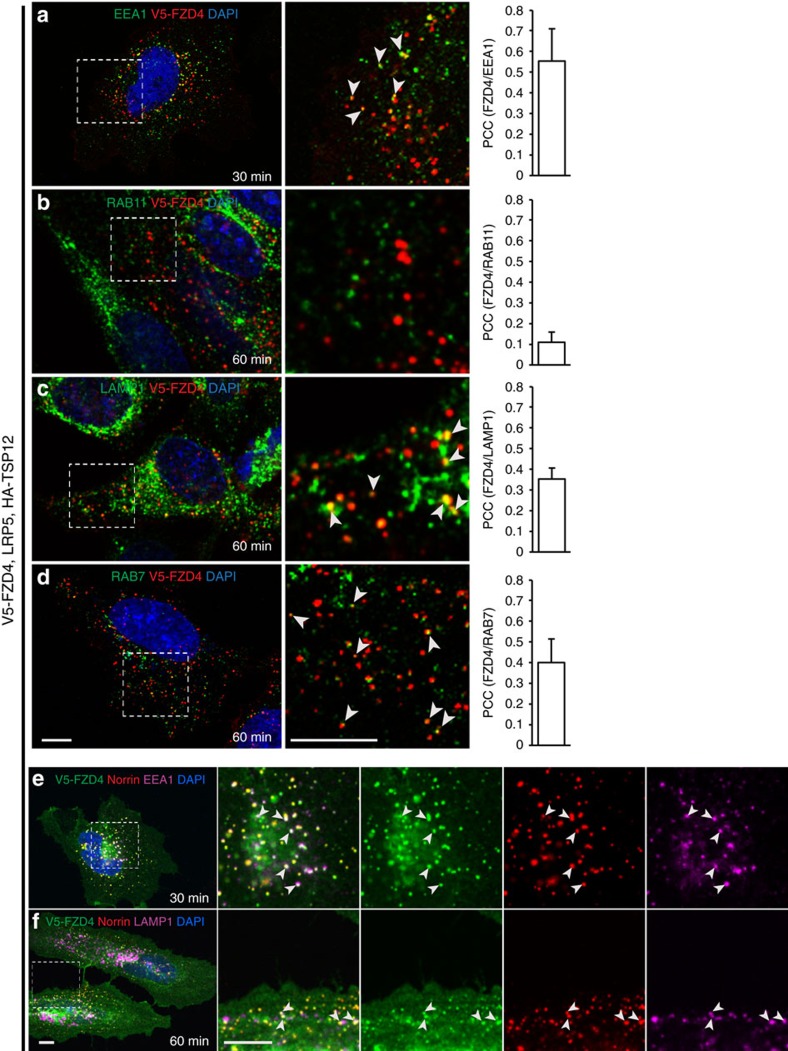
Internalized FZD4 is targeted to the endosome and lysosome. (**a**) 30 min after Norrin-induced internalization at 37 °C FZD4 co-localizes with the early endosomal marker EEA1 in HeLa cells. (**b**) No substantial co-localization with the recycling endosome marker Rab11 was detected. (**c**) FZD4 co-localizes with the lysosomal marker LAMP1 and (**d**) RAB7. Co-localization was quantified using the Pearson correlation coefficient. Mean+s.d. shown, *n*=20. In **a**–**d** internalized FZD4 is specifically revealed by co-internalized V5-antibody. (**e**,**f**) Triple staining for FLAG-AP-Norrin, V5-FZD4 and markers for membrane trafficking excited in the 488 nm (green), 555 nm (red) and 647 nm (magenta) channels. Boxed areas are shown enlarged on the right. The DAPI channel was omitted in the enlarged 3-colour merge. Norrin and FZD4 traffic together into the early endosome and lysosome. Arrowheads show examples of co-localization. Scale bars, 10 μm.

To reveal the destination of the internalized complex, we performed co-localization experiments with markers for endosomal and lysosomal compartments, that is, EEA1 (early endosome) RAB11 (recycling endosome), RAB7 (late endosome) and LAMP1 (late endosome and lysosome). FZD4 puncta were positive for EEA1 15–30 min after induction of endocytosis. At the 60-min time point, FZD4 co-localized in part with EEA1 and in part with RAB7 and LAMP1; virtually no co-localization with RAB11 was detected (Fig. 2a–d). NDP and FZD4 together co-localized with endosomal and lysosomal markers, indicating that receptor and ligand are targeted to the same destination after internalization (Fig. 2e,f).

### TSPAN12 and LRP5/6 are not required for FZD4 endocytosis

We next investigated whether the receptor complex components LRP5/6 or TSPAN12 are required for NDP-induced FZD4 internalization. We used CRISPR/Cas9 mediated genome targeting to generate HeLa cells deficient for both LRP5 and LRP6 and a separate HeLa cell line deficient for TSPAN12 (Supplementary Fig. 2; Supplementary Fig. 3; Supplementary Fig. 4). *LRP5*^−/−^; *LRP6*^−/−^ HeLa cells or *TSPAN12*^−/−^ HeLa cells transfected with FZD4 efficiently internalized NDP into early endosomes (Supplementary Fig. 5a–d). These results indicate that FZD4 is a critical mediator of NDP-induced receptor complex internalization. Although LRP5/6 interacts independently with the endocytic machinery^27^, FZD4 is sufficient to mediate NDP-induced internalization. However, FZD4 alone (in the absence of LRP5/6) is not sufficient to elicit signalling^6^,^9^,^12^.

### NDP-induced FZD4 endocytosis does not require dishevelled

**Figure 3:**
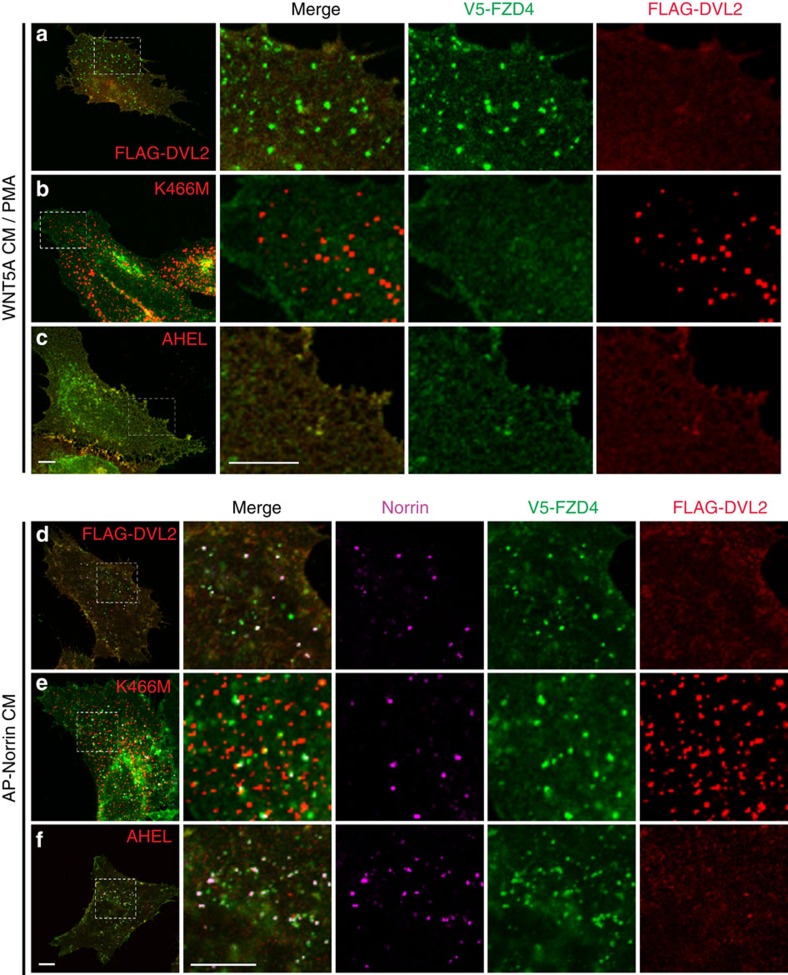
Effect of DVL2 mutations on FZD4 endocytosis. (**a**) WNT5A/PMA-induced FZD4 internalization in HeLa cells. WT DVL2 binds and co-localizes with FZD4 and does not form cytoplasmic aggregates. (**b**) DVL2 K466M is FZD4-binding deficient and thus forms cytoplasmic aggregates. In the DVL2 K466M expressing cells, WNT5A/PMA fails to induce FZD4 endocytosis. (**c**) DVL2 AHEL, which is impaired in AP2 binding, does not form cytoplasmic aggregates but also prevents WNT5A/PMA-induced internalization of FZD4. (**d**–**f**) NDP induces strong FZD4 endocytosis regardless of K466M or AHEL mutations in co-expressed DVL2. Scale bar, 10 μm.

**Figure 4:**
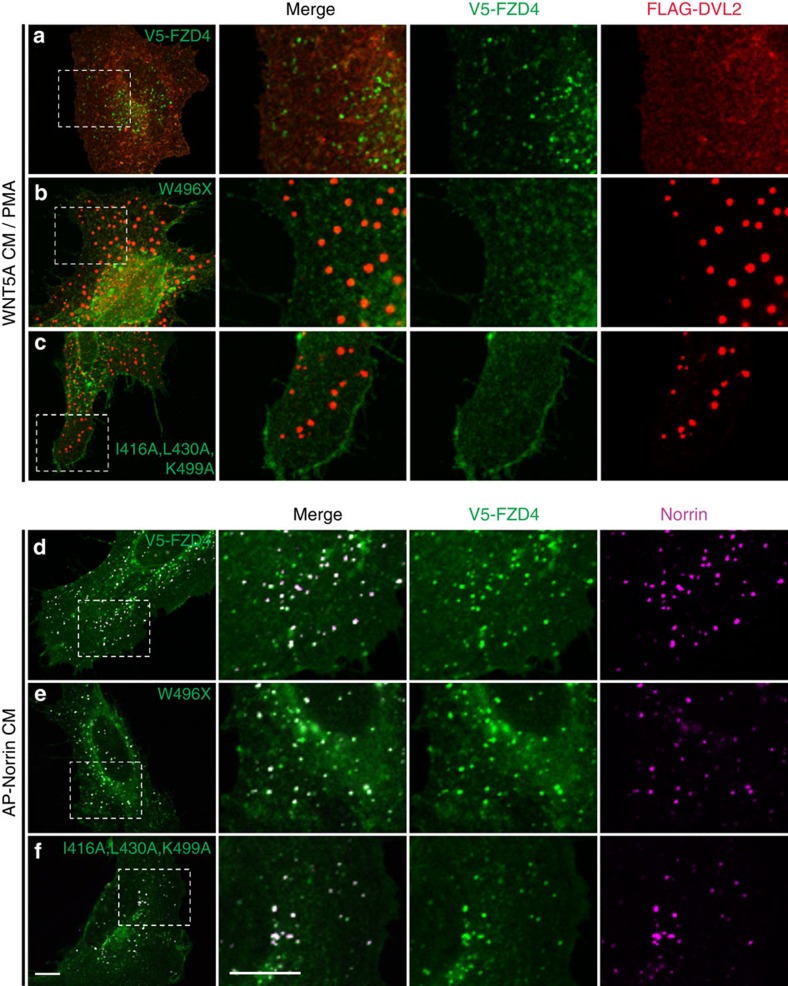
DVL2 binding to FZD4 is dispensable for Norrin-induced endocytosis. (**a**–**c**) FZD4 mutations that disrupt DVL2 binding fail to recruit DVL2 to the plasma membrane and block WNT5A/PMA-induced endocytosis. (**d**–**f**) Norrin-induced FZD4 endocytosis is not affected by FZD4 mutations that disrupt DVL2 binding. DVL2 was co-expressed in all **a**–**f**. Scale bar, 10 μm.

Previous studies showed that the combination stimulus of WNT5A and the phorbol ester PMA triggers FZD4 endocytosis^40^. DVL2 binding to AP2 is essential for this endocytic process, which has been linked to non-canonical signalling^38^. We asked if NDP-induced FZD4 endocytosis also requires DVL2. First, we confirmed that we could trigger FZD4 endocytosis using WNT5A/PMA (Supplementary Fig. 6). We then used previously reported mutations, that is, the K466M mutation in the DEP domain of DVL to disrupt binding to FZD4, and the DVL2 AHEL mutation to disrupt binding to the AP2 μ2 subunit^20^,^38^. We monitored FZD4/DVL2 binding using a DVL aggregation assay^41^. DVL2 formed cytoplasmic aggregates when overexpressed in the absence of FZD4, however, when FZD4 was co-expressed with DVL, aggregates did not form and DVL was efficiently recruited to the plasma membrane (Supplementary Fig. 7c,d). As expected, DVL2 AHEL was recruited to FZD4 in a similar manner as WT DVL2, however, DVL2 K466M remained aggregated in the cytoplasm (Fig. 3, right panels). Both DVL2 AHEL and K466M mutations interfered with WNT5A/PMA-triggered FZD4 endocytosis (Fig. 3a–c). Interestingly, NDP-induced FZD4 endocytosis was not impaired by DVL2 mutations K466M or AHEL (Fig. 3d–f). To further test the requirement of FZD4/DVL2 binding, we mutated residues in the intracellular domain of FZD4 that are predicted to interact with DVL2 based on studies of other Frizzleds. One FZD/DVL interaction site is the KTxxxW motif in the c-terminal tail of FZD4 (ref. 42), and additional interaction sites in intracellular loops have been identified in FZD5 (ref. 41). These studies enabled us to identify a triple mutation — FZD4 I416A, L430A, K499A — that failed to re-localize DVL2 from cytoplasmic aggregates to the plasma membrane. In addition, we found that a FEVR mutation, FZD4 W496X (ref. 43), disrupted DVL2 binding (Supplementary Fig. 7). FZD4 W496X and FZD4 I416A, L430A, K499A failed to internalize in response to WNT5A/PMA stimulation (Fig. 4a–c). In clear contrast, these FZD4 mutations did not interfere with NDP-induced FZD4 endocytosis (Fig. 4d–f).

### NDP-induced FZD4 ubiquitination triggers endocytosis

**Figure 5:**
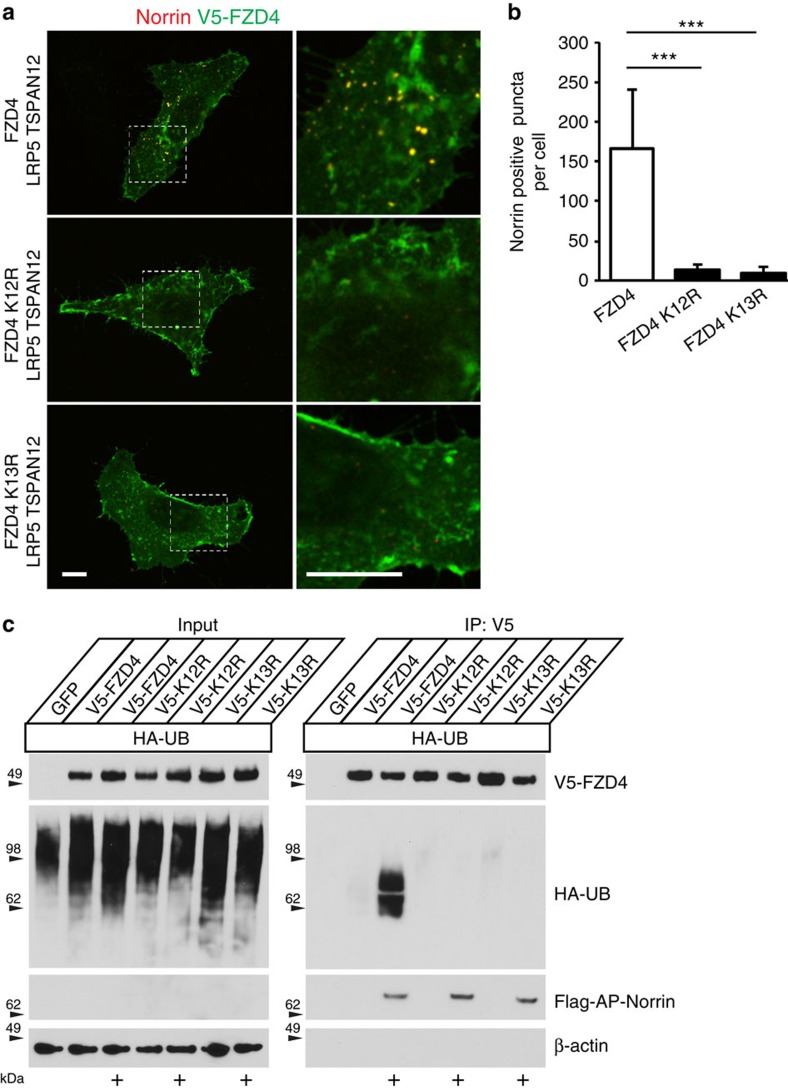
NDP triggers FZD4 endocytosis by promoting FZD4 ubiquitination. (**a**) Norrin-induced FZD4 internalization in HeLa cells. Norin internalization mediated by FZD4 K12R and FZD4 K13R is strongly reduced or absent. Scale bar, 10 μm. (**b**) Quantification of internalized Norrin, mean+s.d., *n*=18, ***P<0.001. (**c**) Intact HeLa cells expressing HA-ubiquitin were incubated with V5 antibody on ice to label cell surface FZD4. At the same time, cells were also incubated with FLAG-AP-NDP conditioned medium or control medium as indicated and subsequently subjected to 20 min internalization at 37 °C. After lysis, cell surface FZD4 was isolated using Protein A/G beads. Wild type FZD4 in the presence of Norrin is ubiquitinated, whereas FZD4 K12R and FZD4 K13R are not ubiquitinated. Note that cell surface bound NDP is below the detection limit in the diluted lysate (input), but can be detected after enrichment in the FZD4 immunoprecipitations.

To test if NDP-induced FZD4 endocytosis requires FZD4 ubiquitination, we used FZD4 K13R (all intracellular lysine residues replaced with arginine, also referred to as K0) and FZD4 K12R (residue 499, which is important for DVL2 binding, is intact). Dishevelled aggregation assays showed that FZD4 K12R retains binding to DVL2 and surface biotinylation assays showed that FZD4 K12R cell surface expression was moderately increased (Supplementary Fig. 8a,b). NDP failed to induce substantial FZD4 endocytosis in cells expressing FZD4 K12R or FZD4 K13R. While the impairment of endocytosis was strong and obvious, inspection of cells in detail and quantification of internalized Norrin positive puncta revealed that endocytosis was not completely abolished (Fig. 5a,b). This mechanism of FZD4 endocytosis was surprising, as FZD4 ubiquitination was previously implicated in regulating receptor clearance, but not in ligand-induced FZD endocytosis^33^,^34^,^36^. To test if NDP triggers FZD4 ubiquitination, HeLa cells were co-transfected with HA-ubiquitin and V5-FZD4. The intact cells were labelled with V5 antibody on ice and cell surface FZD4 was subsequently isolated using Protein A/G beads. To test the role of NDP in promoting ubiquitination, NDP conditioned medium or control conditioned medium was added together with the V5 antibody and cells were subjected to 20 min internalization at 37 °C. NDP-induced a substantial increase of FZD4 ubiquitination, which was blocked by both K12R and K13R mutations (Fig. 5c). To confirm that the mechanisms of FZD4 endocytosis are recapitulated in endothelial cells, we used a CNS endothelial cell line, bEND.3. Internalized NDP co-localized with FZD4, EEA1 and LAMP1, and internalization was prevented by K12R mutations in FZD4 (Supplementary Fig. 8c–g). Together, our results reveal that NDP promotes both FZD4 ubiquitination and FZD4 internalization.

### Blockade of endo-lysosomal trafficking inhibits signalling

**Figure 6:**
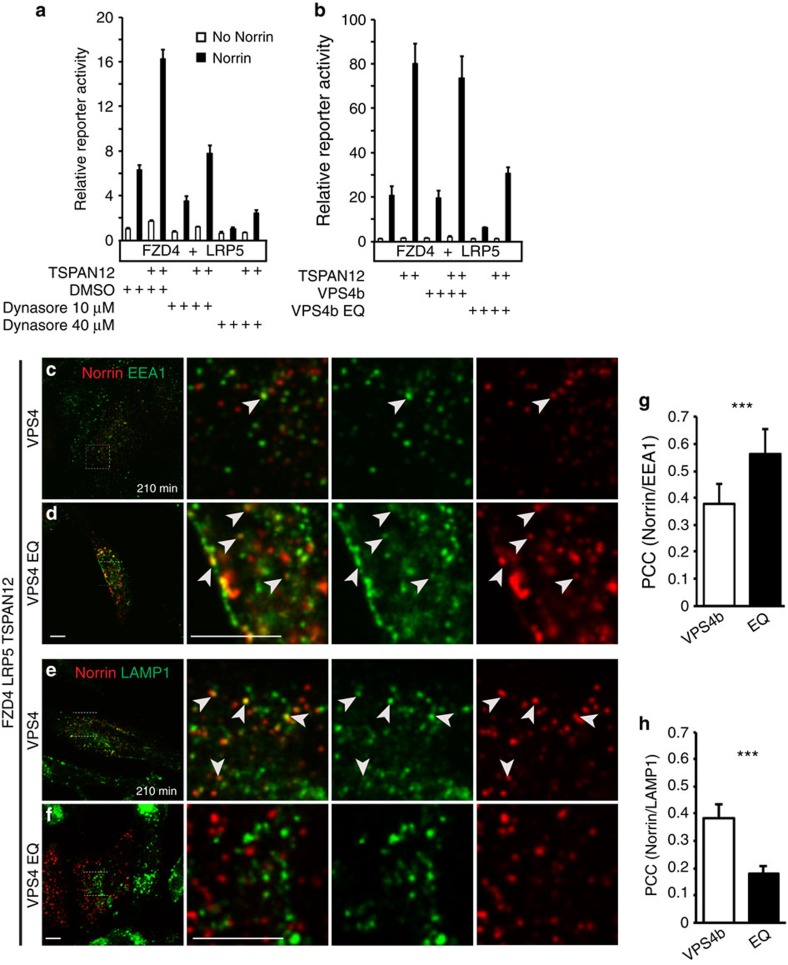
Inhibition of endo-lysosomal trafficking impairs FZD4 signalling. (**a**) TOPFlash reporter assays in 293T cells transfected with the indicated constructs show that Dynasore inhibits Norrin/FZD4 signalling in a dose-dependent manner. (**b**) Co-expression of WT VPS4 has no effect on Norrin/FZD4 signalling, whereas dominant negative VPS4 EQ impairs Norrin/FZD4 signalling. Data are normalized to the first bar in each graph (*n*=3, mean+s.d. shown). Different signalling strength in **a** versus **b** is due to a shorter stimulation time in experiments where dynasore was added. (**c**–**f**) 3.5 h after induction of endocytosis, Norrin co-localized predominantly with LAMP1 in VPS4 expressing HeLa cells, and predominantly with EEA1 in VPS4 EQ expressing cells. In VPS4 EQ expressing cells, Norrin accumulation in early endosomes produced occasionally unusually large fluorescent puncta. (**g**,**h**) Quantification of co-localization (*n*=20, mean+s.d. shown, ***P<0.001). Scale bar, 10 μm.

To determine the functional role of endocytosis and endo-lysosomal trafficking in NDP/FZD4 signalling, we blocked these processes using inhibitors and dominant negative interference, and examined the resulting signalling responses using the TOPFlash reporter assay^44^. Dynasore is a small molecule inhibitor of dynamin, a GTPase that is required for clathrin- and caveolin-dependent endocytosis. We found that dynasore inhibited NDP-induced signalling in a concentration-dependent manner. TSPAN12 is a ligand-specific co-activator in NDP/FDZ4 signalling^9^, and Dynasore did not affect TSPAN12-mediated enhancement of signalling relative to the base level (Fig. 6a). We investigated potential crosstalk of NDP with endogenous WNTs and found that (i) NDP and WNT7B did not show additive effects in TOPFlash assays, (ii) NDP-induced signalling was not blocked by the combination of two WNT inhibitors, sFRP1 and IWP-2, and (iii) Dynasore inhibited NDP-induced signalling even when endogenous WNTs were inactivated by sFRP1 and IWP-2 treatment (Supplementary Fig. 9a–c). These results confirmed that Dynasore affected NDP/FZD4 signalling in a largely Wnt-independent manner.

ESCRT complexes function in the MVB pathway to deliver ubiquitinated membrane proteins to the late endosome. Multiple components of ESCRT-0 and ESCRT-I complexes bind ubiquitinated cargo via ubiquitin interacting motifs. The ATPase VPS4 is an essential mediator of MVB formation by disassembling ESCRT-III complexes and recycling individual components for subsequent rounds of vesicle formation^45^. Consistent with previous reports^30^, we found that the co-transfection of the ATPase VPS4b (with c-terminal mycHis tag) did not affect NDP/FZD4 signalling, however, the dominant negative VPS4b EQ, that is, E235Q in mouse VPS4b (ref. 46), strongly inhibited signalling (Fig. 6b). On the subcellular level, VPS4 EQ affected NDP trafficking through the endo-lysosomal compartment. 3.5 h after induction of endocytosis, NDP had moved predominantly to the LAMP1-positive compartment in VPS4 expressing cells, whereas NDP still substantially co-localized with EEA1 and showed minor co-localization with LAMP1 in VPS4 EQ expressing cells (Fig. 6c–h). Together, these data indicate that impaired trafficking through the MVB pathway and NDP/FZD4 signalling defects are associated.

### Angiogenesis and barrier defects in VPS4 EQ expressing mice

**Figure 7:**
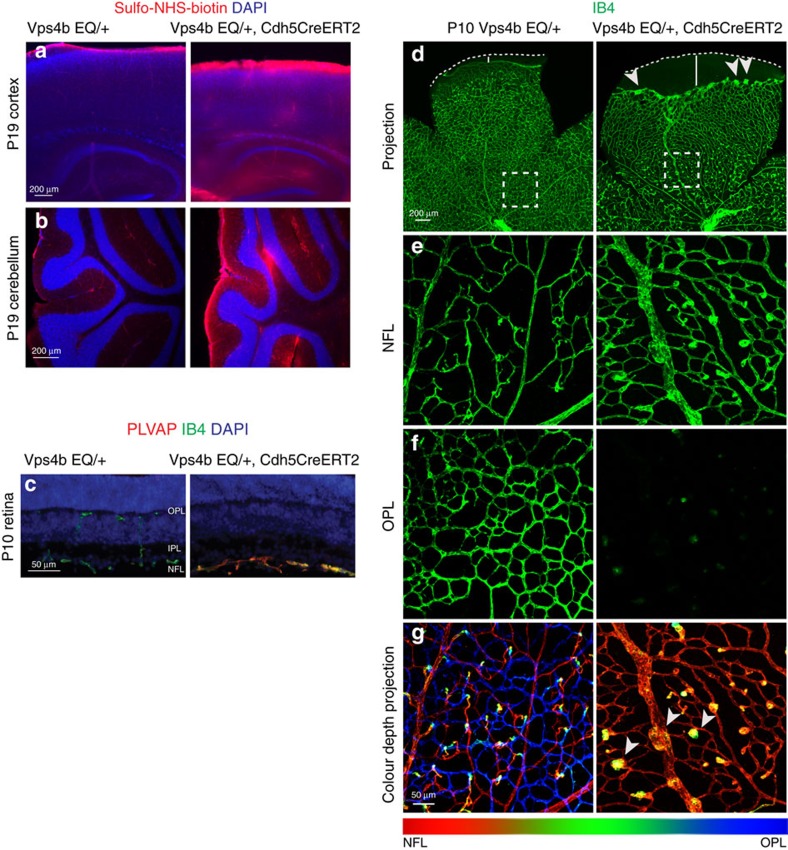
Inhibition of endo-lysosomal trafficking in vascular endothelial cells *in vivo*. (**a**) Blood-CNS barrier defects are revealed in the cortex and hippocampus of Cre-activated *Rosa*26 LSL-VPS4 EQ mice transcardially perfused with Sulfo-NHS-biotin by staining with Streptavidin coupled to Alexa 555. (**b**) Barrier defects in the molecular layer of the cerebellum. (**c**) Retinal sections show failure of intraretinal vascularization and strongly increased PLVAP expression in VPS4 EQ expressing mice. These defects recapitulate hallmark phenotypes of impaired Norrin/FZD4 signalling in the retinal vasculature. NFL, nerve fiber layer; IPL, inner plexiform layer; OPL, outer plexiform layer. (**d**) Confocal projections of the retinal vasculature in P10 whole mount retinas stained with IsolectinB4-Alexa488. Characteristic Norrin LOF phenotypes are observed. Progression towards the rim of the retina (indicated by white dashed line) is delayed (distance from the vascular front to the rim of the retina indicated by white bar) and the microscopic appearance of the vascular front is altered (arrowheads). (**e**,**f**) Optical sections of the boxed areas in **d** show separate representations of the vascular plexuses located at different depth levels of the retina. Normal vasculature is present in the nerve fiber layer (NFL) and outer plexiform layer (OPL) in control mice, whereas intraretinal capillaries in the OPL of VPS4 EQ expressing mice are absent. (**g**) Colour depth projections show the vascular beds of the boxed areas in **d** as single projection, in which distinct depth levels are colour coded. These projections reveal the characteristic glomeruloid vascular malformations (yellow structures, arrowheads) in the nerve fiber layer vasculature, similar to the defects described in *Fzd4*, *Ndp*, *Lrp5* and *Tspan12* mutant mice.

To further test the requirement of moving ubiquitinated cargo through the MVB pathway in NDP/FZD4 signalling, we constructed a Cre-activated knock-in allele that encodes murine VPS4 EQ and targeted it to the *Rosa26* locus (Supplementary Fig. 10). In this allele, the CAG promoter is separated from the open reading frame by multiple transcriptional stop signals, which in turn are flanked by loxP sites (loxP-STOP-loxP, LSL). We used an endothelial cell specific Cre-ERT2 driver line, Tg(Cdh5-cre/ERT2)1Rha (ref. 47) to induce VPS4 EQ expression by tamoxifen injection at P6 and P7. At P9, robust VPS4 EQ expression was detected in lysates of lung tissue, which was used because of its high vascular density (Supplementary Fig. 10c). Analysis of P19 sagittal brain sections revealed widespread leakage of Sulfo-NHS-biotin tracer in VPS4 EQ activated mice, including in the cortex, hippocampus, and molecular layer of the cerebellum (Fig. 7a,b). P10 retinal sections of VPS4 EQ activated mice displayed the characteristic endothelial cell PLVAP^high^ expression signature and showed that the formation of intraretinal blood vessels had failed (Fig. 7c). P10 whole mount retinas were analyzed using confocal microscopy and showed that VPS4 EQ expressing mice displayed additional characteristic loss-of-function phenotypes indicative of impaired NDP/FZD4 signalling^9^,^12^,^13^,^14^,^15^. The radial vascular growth in the nerve fiber layer was delayed, vascular malformations were detected at the vascular front, and blood vessels failed to invade the outer plexiform layer of the retina. Highly characteristic glomeruloid vascular malformations formed instead of intraretinal capillaries (Fig. 7d–g). VPS4 EQ expression resulted in sudden lethality 4–5 days after recombination, reminiscent of the lethality reported for postnatal loss of β-catenin signalling in endothelial cells^6^. Together, these data indicate that transport of ubiquitinated cargo through the endo-lysosomal compartment is required for β-catenin signalling in endothelial cells and is essential for CNS angiogenesis and blood-CNS barrier formation.

## Discussion

The ESCRT-dependent MVB pathway is implicated in GSK3ß sequestration and canonical Wnt signalling^29^,^30^, however, the mechanism of ligand-induced receptor internalization remain unclear. A central conclusion from the present study is that NDP triggers FZD4 endocytosis by promoting FZD4 ubiquitination. Internalized FZD4, in complex with associated molecules LRP5 and TSPAN12 and continuously bound ligand, is then transported to the endosome and lysosome. The internalization of the receptor complex generates a component of signalling that is blocked by perturbing the MVB pathway. A second major conclusion is that blocking the movement of ubiquitinated cargo into late endosomes and lysosomes causes CNS angiogenesis and blood-CNS barrier phenotypes that are characteristic for impaired β-catenin signalling. This finding raises the possibility that CNS vascular diseases, including vascular malformations and diseases associated with an impaired blood-brain barrier, may be linked to dysfunctional endo-lysosomal trafficking.

We further show that the NDP/FZD4 ligand-receptor pair provides a suitable model system to study FZD internalization in β-catenin-dependent signalling. A challenge to biochemical and cell biological studies using Wnts is the promiscuous interaction of Wnt ligands with multiple FZD receptors and the requirement for lipid modifications, which enable the secretion of Wnts^48^ but make handling Wnts more difficult. In comparison, NDP signals specifically through FZD4, is genetically well characterized, and does not require lipid modification. Both internalized ligand and receptor can be readily localized within cells. Thus, NDP/FZD4 signalling provides a system to investigate mechanisms of ligand-induced FZD internalization in canonical signalling.

Previous studies on ligand-induced receptor internalization have predominantly focused on the ligands WNT5A in non-canonical signalling and WNT3A in canonical signalling. We find that NDP-induced FZD4 internalization does not require DVL2/FZD4 binding, whereas WNT5A/PMA-induced FZD4 internalization requires this interaction. Studies on WNT3A-induced receptor internalization have characterized mechanisms of LRP6 endocytosis^27^, but little is known about interactions of FZD with the endocytic machinery when internalization is induced by canonical ligands. In this study we identified ubiquitinated FZD4 as key interaction partner with the endocytic machinery in NDP-induced internalization. Although FZD4 co-internalizes with LRP5/6, neither LRP5/6 nor DVL are required for internalization. NDP-induced endocytosis of FZD4 without associated LRP5/6 or DVL (for example, in LRP5/6 DKO cells or when FZD4 and DVL cannot interact due to mutations in FZD4 or DVL) is, however, not sufficient to elicit signalling. FZD and LRP5/6 appear to have separate interactions with mediators of internalization, and the contribution of FZD in the process of ligand-induced endocytosis had so far been unclear.

FZD receptor levels and signalling strength are controlled by E3 ligases ZNRF3 and RNF43, which are in turn modulated by R-spondin ligands and LGR receptors^34^,^36^. Here, we find that FZD4 ubiquitination is strongly promoted by NDP and leads to NDP-induced endocytosis of FZD4. An important distinction in the regulation of FZD receptor levels and NDP-induced FZD4 internalization is the ability to inhibit the destruction complex. Clearance of FZD in the absence of WNT/NDP does not elicit signalling. In contrast, NDP-induced internalization of a complex containing FZD4, NDP and LRP5 appears to be positively coupled to a component of signalling through inhibition of the destruction complex. NDP has been reported to bind LGR4 (ref. 49) and thus, next to its direct actions via FZD4 binding, may also promote FZD signalling in an indirect (that is, R-spondin-like) manner, by removing negative regulators RNF43 and ZNRF3 from the cell surface.

Previous reports indicated that FZD4 ubiquitination is not induced by WNT3A^33^. Ligand properties such as the specific affinity of the ligand for FZD4 (versus other FZDs) may account for different results using NDP versus WNT3A stimulation. In addition, WNT-induced internalization may require additional receptor complex components that stabilize ligand-receptor interactions, a candidate for such a function is GPR124 (ref. 50).

The dominant negative form of VPS4 was previously shown to inhibit Wnt/β-catenin signalling in cell-based assays and in frog axis duplication assays^30^. We confirmed and extended these observations in the context of CNS vascular biology. VPS4 is essential for multivesicular body formation and fulfills important roles in sorting ubiquitinated cargo into late endosomes. The blood-brain barrier phenotypes we observed upon activation of dominant negative VPS4 EQ in endothelial cells are similar (albeit probably milder) to those reported for conditional disruption of the β-catenin gene in endothelial cells^6^. Retinal phenotypes of VPS4 EQ expressing mice are clearly overlapping with defects in mice, in which NDP/FZD4 signalling is disrupted (see Introduction). These striking phenotypic similarities include the characteristic upregulation of the endothelial cell fenestration component PLVAP, delayed progression of the vascular front in the superficial vascular plexus, specific failure of *intra*-retinal capillary development, and glomeruloid vascular malformations. Impaired endo-lysosomal trafficking could also impair additional signalling systems, for example GSK3β activity that is not mediated by β-catenin (WNT/STOP signalling^51^), or distinct angiogenic signalling systems, for example, Notch, VEGFR2 or Eph/Ephrin signalling^52^. However, we observed no retinal phenotypes in VPS4 EQ expressing mice that were not also observed in the mouse models with impaired NDP/FZD4 signalling. The specific phenotypic profile in the absence of broader vascular phenotypes suggest that (i) VPS4 EQ activation impairs canonical signalling in a surprisingly specific manner, or (ii), that impaired β-catenin signalling is one of the earliest consequences of VPS4 EQ activation in our short-term *in vivo* experiments, or (iii), that impairments in other angiogenic pathways synergize with impaired β-catenin signalling. This would be expected if the additional impaired pathway mediates the expression of genes that are required for β-catenin signalling.

Mutations in human genes *NDP*, *FZD4*, *LRP5* and *TSPAN12* cause familial exudative vitreoretinopathy, a potentially blinding disease initially characterized by retinal hypovascularization^16^. Our results suggest a link between defective endo-lysosomal trafficking and FEVR and raise the possibility that mediators of membrane dynamics are additional unknown FEVR genes or modifiers of FEVR expressivity. In neurology, blood-brain barrier defects have been connected to neurodegenerative diseases, including Alzheimer’s disease and Parkinson’s disease^7^. Thus, in light of our findings, vascular endo-lysosomal trafficking defects may emerge as factors in blood-CNS barrier dysfunction and neurodegenerative diseases.

## Methods

### DNA constructs

Super 8X TOPFlash^44^ and CMV-Renilla were kindly provided by Dr Michael Klymkowsky. The following plasmids were obtained from Addgene: 3xFLAG-DVL2 (deposited by Dr Jeff Wrana), pRK5-HA-Ubiquitin-WT (deposited by Dr Ted Dawson), sFRP-1-CS2+ (deposited by Dr Randall Moon). We obtained Image clones encoding hLRP5 (Image 9056965), hFZD4 (Image 5199771), hNDP (Image 5179578) and subcloned the coding sequences into pcDNA3.3 TOPO (Invitrogen) using PCR. Constructs contained a HSV-1 glycoprotein D signal sequence (ss) as indicated. pcDNA3.3 ss.V5-FZD4 (aa37-537), pcDNA3.3 ss.FLAG-AP-hNorrin (aa25-133), pAPtag5 ss.AP-hNorrin-mycHis (aa25-133), pcDNA3.3 ss.3xFLAG-hLRP5 (aa32-1615), pcDNA3.3 ss.HA-hLRP5 (aa32-1615), pcDNA3.3 hLRP5 (aa1-1615), pcDNA3.3 hWNT3A, and pcDNA3.3 HA-hTSPAN12 were verified to be fully functional in TOPFlash assays. pcDNA3.3 V5-FZD4 K13R was generated by subcloning a DNA fragment obtained by gene synthesis (Biobasic), in which all intracellular lysine resides were mutated to arginine, pcDNA3.3 V5-FZD4 K12R (with intact lysine in position 499) and pcDNA3.3 V5-FZD4 M157V were generated by site directed mutagenesis (Quikchange, Stratagene).

### Cell culture

293T cells (ATCC CRL-3216), HeLa cells (ATCC CCL-2), or bEND.3 cells (ATCC CRL-2299 (ref. 53)), were cultured in high glucose DMEM with 10% FBS (full DMEM) at 37 °C in the presence of 5% CO_2_. For maintenance, cells were split 1:6 at near confluence using 0.05% Trypsin-EDTA. Mycoplasma contamination was excluded by PCR.

### Generation of conditioned medium

The concentration of NDP in the conditioned medium (CM) was significantly increased when NDP was fused to AP. Both FLAG-AP-NDP and AP-NDP-mycHis constructs/CM were fully functional in TOPFlash assays, but allowed for different modes of detection (the detection of FLAG via the M2 antibody is more sensitive than the detection of myc with the 9E10 antibody). AP-NDP CM was generated by transient transfection of 293T cells for 3–4 days. Acidity was controlled by adding sterile 1 M HEPES pH 8.0. CM was filtered and stored at 4 °C or frozen. WNT5A CM was generated from L Wnt-5A cells (ATCC CRL-2814) stably expressing WNT5A. Cells were maintained in the presence of G418 and CM was collected twice for 2 days without G418. Immunoblotted WNT5A was probed using rabbit anti WNT5A antibody (1:1,000, Cell Signaling, #2392).

### Stimulation of endocytosis

Twenty thousand HeLa cells were seeded into 8-well chamber slides (Labtek II, Nunc) and transfected 3–6 h later with a total of 160 ng DNA (typically: 25 ng V5-FZD4, 50 ng LRP5, 50 ng HA-TSPAN12, 35 ng empty vector using TransIT LTI (Mirus). 36 h later, cells were placed on ice and incubated for 1 h with ice cold FLAG-AP-NDP or AP-NDP-mycHis CM. For quantitative NDP internalization assays CM was diluted 1:10 with full DMEM. Cells were washed once with ice cold full DMEM and placed in a tissue culture incubator at 37 °C for 30 min or the otherwise indicated period of time. Subsequently, cells were washed one time for 3 min at RT with 150 mM NaCl, 50 mM glycine, pH 2.4, and then rinsed twice with PBS before fixation with 10% NBF for 5 min at RT. Where endocytosed FZD4 was visualized by FZD4 pre-labelling (‘antibody feeding’), intact cells on ice were incubated with both NDP CM and ms anti V5 (Serotec, 1:2,000) on ice for 1 h, then internalization was triggered at 37 °C for 30 min. Alternatively, FZD4 endocytosis was triggered with prewarmed WNT5A CM and 1 μM PMA (from a 1,600 × stock in DMSO) for 40 min in a cell culture incubator at 37 °C (ref. 40). Control cells were incubated with medium containing the same concentration of DMSO. Cells were washed with PBS and fixed for immuno-staining.

### Immunofluorescence staining and confocal imaging of cultured cells

After fixation with 10% NBF for 10 min at RT, cells were rinsed twice with PBS and blocked/ permeabilized with PBS, 5% goat serum, 0.1% Triton X-100 for 20 min at RT. Cells were incubated with primary antibodies in blocking buffer for 1 h at RT, washed three times with PBS, 0.1% Triton X-100, and incubated with secondary antibody in blocking buffer for 1 h at RT. After staining, cells were rinsed three times in PBS, 0.1% Triton X-100 and mounted using Fluoromount G. When two mouse (ms) primary antibodies were used, a three-step protocol was used, that is, the third step was incubation with a ms primary antibody directly conjugated to a fluorophore (for example, ms anti FLAG to detect NDP in the 555 nm channel, combined with ms V5-Alexa-488 staining as a third step, to detect FZD4). In some experiments triple immunostain was performed using secondary antibodies conjugated to Alexa 488, Alexa 555, and Alexa 647. Primary antibodies were: ms anti V5 (1:2,000, Serotec, clone SV5-PK1, in some experiments this antibody was conjugated to Alexa 488 and used at 1:200), ms anti FLAG (1:1,000, Sigma, clone M2), rat anti HA (1:500, Roche, clone 3F10), mouse anti myc (1:1,000, Thermo, clone 9E10). The following rabbit antibodies were from Cell Signaling: anti myc (1:200, clone 71D10) EEA1 (1:200, clone C45B10), RAB5 (1:200, clone C8B1), RAB7 (1:100, clone D95F2), RAB11 (1:200, clone D4F5), LAMP1 (1:200, clone D2D11). Fluorescence was visualized at RT using a Zeiss LSM510 (63 × oil) or a Nikon A1 (100 × oil) confocal laser scanning system. Co-localization analysis was performed using the Pearson’s correlation coefficient method in NIS-Elements. NDP positive puncta were quantified using NIS-Elements.

### CRISPR/Cas9-mediated gene targeting

HeLa cells were transfected with plasmids encoding Cas9 and sgRNAs (deposited by Dr Feng Zhang: addgene #42230, co-transfected with GFP, or #62988, selected with puromycin). Individual colonies derived from single cells were picked and expanded. HeLa cell clones were screened by genomic PCR. PCR products were analyzed after blunt ligation into pJet2.1 by sequencing of 10 individual bacterial clones. Clones were identified as positive if targeted alleles were null alleles and if a maximum of two types of targeted alleles was found. sgRNA sequences are shown in Supplementary Figures.

### TOPFlash luciferase assay

293T cells were seeded by dispensing 0.4 ml of a 300 K ml^−1^ cell suspension into 48-well plates. 3–6 h later cells were transfected by combining 20 μl Optimem with 160 ng DNA per well and 20 μl Optimem with 0.32 μl of Trans-IT LT1 (Mirus). After 15 min complex formation at RT the transfection mix was added to the cells. Per well, transfections contained the following plasmids: 4 ng FZD4, 8 ng LRP5, 8 ng pcDNA 3.3 HA-TSPAN12 (or 8 ng pcDNA 3.3 EGFP as control), and 140 ng of reporter mix. 20 ng Vps4b was co-transfected in some experiments. Reporter mix had a concentration of 1 mg ml^−1^ and was composed of TOPFlash plasmid, CMV-Renilla, and Lef1 in a ratio of 7:2:1. Six hours after transfection, cells were stimulated with 125 ng ml^−1^ recombinant NDP (R+D Systems) for 12–16 h. Dual-Glo luciferase assays (Promega) were performed in white, flatbottom, non-treated polystyrene 96-well plates following the manufacturer's instructions. IWP-2 was used at a final concentration of 5 μM from cell seeding onward. In experiments where dynasore was added, cells were allowed to express for 17 h, were then grown with dynasore for 1 h and subsequently with NDP for additional 4 h. 20 mM or 5 mM dynasore stock solutions in DMSO were diluted 1:500 into a 48-well containing 500 μl medium, DMSO in the same dilution was added to control wells. Unless otherwise noted, data were analyzed by calculating the ratio of firefly/renilla luciferase signals and normalizing the data to the data point shown on the left of each bar graph.

### Animals

All animal use for this study was approved by the Institutional Animal Care and Use Committee (IACUC) at the University of Colorado Boulder. Male or female animals on a C57BL/6J background were used for experiments at postnatal ages P10 or P19. Tg(Cdh5-cre/ERT2)1Rha mice were provided by Dr. Ralf Adams^47^ and genotyped by PCR using the following primer set: 5′-GCCTGCATTACCGGTCGATGC-3′/5′-CAGGGTGTTATAAGCAATCCC-3′. Cre-activatable Vps4b EQ mice were generated by a knock-in strategy into the *Rosa*26 locus. Mouse Vps4bEQ cDNA with MycHis tag and bovine growth hormone polyadenylation sequence was amplified from pEGFP-N1-mVps4b-EQ-MycHis plasmid (gift from Dr Phyllis Hanson) and subcloned into NotI/EcoRI sites of transfer vector pMAT1. The Vps4b EQ sequence with pMAT1 encoded portions of the CAG promoter and lox-STOP-lox sequence was subcloned into the MreI and EcoRI sites of pHW249 targeting vector (pMAT1 and pHW249 were kindly provided by Dr Jeremy Nathans). The targeting construct was linearized with KpnI before electroporation into EC7.1 ES cells in the University of Colorado Anschutz Medical Campus gene targeting core. Positive ES cell clones were identified by PCR using primers upstream and downstream of the 5′ arm (5′-AAGAAGAGGCTGTGCTTTG-3′/5′-TTGGCGTTACTATGGGAAC-3′) as well as using primers upstream and downstream of the 3′ arm (5′-GAACCAGCTGGGGCTCGACTAGAG-3′/5′-CAAGCACTGTCCTGTCCTCAAG-3′). Selected positive clones were karyotyped and two correct clones were injected into blastocyst to generate chimeric mice. Chimeric mice were crossed with C57BL/6J mice and genotyped for the presence of a 483 bp *Vps4b* amplicon using the primers

5′-TGGAGATAAACTCTTGGAGCC-3′/5′-CCGGAATTCTCCGCCTCAGAAGCCATAGAGC-3′. No randomization or blinding was used in animal studies. Three or more animals per genotype were analyzed.

### Preparation and administration of tamoxifen

Tamoxifen (Sigma) solutions were freshly prepared at 1 mg ml^−1^ in sterile corn oil (Sigma) the day before each injection using a nutator to facilitate dissolution overnight at RT. Solutions were sterile filtered before injection. 50 μl of 1 mg ml^−1^ tamoxifen in corn oil was administrated by intraperitoneal injection at P6 and P7 for retinal analysis at P10. For the analysis of P19 brains, 100 μl 20 mg ml^−1^ tamoxifen was injected at P16 and P17.

### Immunofluorescence staining of tissue

P19 deeply anesthetized mice were transcardially perfused with 20 ml 0.3 mg ml^−1^ Sulfo-NHS-Biotin (Thermo 21335) in PBS and 10 ml 2.5% NBF. Saggital brains sections were collected at 50 or 150 μm thickness using a cryostat or vibratome, respectively. Tissues were fixed for 3 h in 10% neutral buffered formalin (NBF) on ice. For the generation of retinal sections, the cornea of fixed eyeballs was incised before specimens were cryoprotected in 30% sucrose, PBS at 4 °C overnight. Specimens were embedded in O.C.T. compound (Tissue-Tek), and rapidly frozen. PBS, 0.1% Triton X-100 (PBST) with 5% goat serum was used for blocking for 40 min at RT, primary antibodies diluted in blocking buffer were applied overnight at 4 °C. Slides were washed with 0.1% PBST 3 times for 2 min. Secondary antibody in blocking buffer was applied for 1 h at RT, slides were washed 3 times for 2 min in 0.1% PBST before mounting. For whole mount retina immunostaining^54^, tissues were fixed for 3 h in 10% neutral buffered formalin (NBF) on ice. Retinas were blocked for 2–4 h at RT in PBST 0.5%, 5% goat serum, and incubated overnight at 4 °C with primary antibody in blocking buffer. After washing 5 times for 1 h at RT with 0.5% PBST, specimens were incubated with secondary antibody and DAPI (1 μg ml^−1^) in blocking buffer at 4 °C. The following primary antibodies and lectins were used for this study: GS Isolectin B4 Alexa-488 conjugate 1:100 (Invitrogen, I21411), rat PLVAP 1:200 (BD Biosciences, 550563). Biotin was detected using Alexa-555 coupled Streptavidin (1:1,000, Invitrogen). Fluorescence was visualized using a Zeiss LSM510 confocal laser scanning system or Leica DM IL epifluorescent microscope.

### Ubiquitination assay

HeLa CCL-2 cells in 10 cm^2^ dishes were co-transfected with 3 μg of HA-Ubiquitin, 1 μg of V5-FZD4 (wild type, V5-FZD4-K12R, or V5-FZD4-K13R), and 5 μg of EGFP plasmids using 54 μl of 1 mg ml^−1^ linear PEI, MW 25,000 (Polysciences). After 20–24 h, cells were placed on ice and growth medium was removed. Samples were incubated for 1 h on ice with 5.5 ml FLAG-AP-NDP conditioned medium (CM) or growth medium, each supplemented with 1 μg ms anti-V5 (AbD Serotec). Cells were then incubated at 37 °C for 20 min to induce internalization. CM was removed and cells were washed twice with ice cold 1x PBS. Cells were then lysed with 4 ml lysis buffer (150 mM NaCl, 50 mM Tris pH 8.0, 1 mM EGTA, 1% NP40, 0.1% N-dodecyl-beta-D-maltoside, 3 mM MgCl_2,_ DNAseI 80 units per ml (Roche), and 1 × complete ultra EDTA-free protease inhibitor cocktail (Roche)), on ice for 40 min with gentle shaking. Cell debris and unsolubilized material were pelleted at 20,000*g* centrifugation at 4 °C for 45 min. For the analysis of input from each sample, 104 μl of the supernatant were removed and combined with 40 μl 4 × LDS sample buffer (Invitrogen) and 16 μl 1M DTT. 3.75 ml of remaining supernatant were incubated with 30 μl ProteinA/G beads (Pierce) at 4 °C for 45 min with gentle rocking. Samples were transferred into spin columns with screw cap (Pierce), which were attached to a vacuum manifold (Qiagen) and washed 5 times with 0.7 ml wash buffer (150 mM NaCl, 50 mM Tris pH 8.0, 1 mM EGTA, 1% NP40, 3 mM MgCl_2_) at RT by applying vacuum. V5-FZD4 and associated proteins were eluted from the beads by incubating the plugged and capped columns with 120 μl 1 × LDS sample buffer (Invitrogen) containing 100 mM DTT for 20 min at 45 °C in a thermomixer (Eppendorf) with rapid shaking. Eluates were spun through the column into collection tubes. 45 μl of the eluate were loaded onto SDS–PAGE gels. Anti-HA-HRP (1:1,000, Roche), anti-V5-HRP (1:10,000, AbD Serotec), and anti-FLAG-HRP (1:1,000, Sigma) were used to probe immunoblots.

### Software and figure preparation

Zeiss Zen 2011, Nikon NIS-Elements, ImageJ, Microsoft Office 2016 and Adobe Photoshop/Illustrator CS6 were used for data analysis and figure preparation. Full scans of immunoblots are shown in Supplementary Fig. 11.

### Statistics

Averages were calculated from triplicates. Groups were compared using a two-tailed, unpaired Student’s *t*-test. *P*-values<0.05 were considered significant.

### Data availability

All relevant data are available from the authors.

## Additional information

**How to cite this article:** Zhang, C. *et al*. Norrin-induced Frizzled4 endocytosis and endo-lysosomal trafficking control retinal angiogenesis and barrier function. *Nat. Commun.* **8,** 16050 doi: 10.1038/ncomms16050 (2017).

**Publisher’s note:** Springer Nature remains neutral with regard to jurisdictional claims in published maps and institutional affiliations.

## Supplementary Material

Supplementary Information

## References

[b1] Clevers H. & Nusse R. Wnt/beta-catenin signaling and disease. Cell 149, 1192–1205 (2012). 22682243 10.1016/j.cell.2012.05.012

[b2] Liebner S. . Wnt/beta-catenin signaling controls development of the blood-brain barrier. J. Cell Biol. 183, 409–417 (2008). 18955553 10.1083/jcb.200806024 PMC2575783

[b3] Stenman J. M. . Canonical Wnt signaling regulates organ-specific assembly and differentiation of CNS vasculature. Science 322, 1247–1250 (2008). 19023080 10.1126/science.1164594

[b4] Daneman R. . Wnt/beta-catenin signaling is required for CNS, but not non-CNS, angiogenesis. Proc. Natl Acad. Sci. USA 106, 641–646 (2009). 19129494 10.1073/pnas.0805165106 PMC2626756

[b5] Wang Y. . Norrin/Frizzled4 signaling in retinal vascular development and blood brain barrier plasticity. Cell 151, 1332–1344 (2012). 23217714 10.1016/j.cell.2012.10.042 PMC3535266

[b6] Zhou Y. . Canonical WNT signaling components in vascular development and barrier formation. J. Clin. Invest. 124, 3825–3846 (2014). 25083995 10.1172/JCI76431 PMC4151216

[b7] Zhao Z., Nelson A. R., Betsholtz C. & Zlokovic B. V. Establishment and dysfunction of the blood-brain barrier. Cell 163, 1064–1078 (2015). 26590417 10.1016/j.cell.2015.10.067 PMC4655822

[b8] Ye X., Wang Y. & Nathans J. The Norrin/Frizzled4 signaling pathway in retinal vascular development and disease. Trends Mol. Med. 16, 417–425 (2010). 20688566 10.1016/j.molmed.2010.07.003 PMC2963063

[b9] Junge H. J. . TSPAN12 regulates retinal vascular development by promoting Norrin- but not Wnt-induced FZD4/beta-catenin signaling. Cell 139, 299–311 (2009). 19837033 10.1016/j.cell.2009.07.048

[b10] Chang T. H. . Structure and functional properties of Norrin mimic Wnt for signalling with Frizzled4, Lrp5/6, and proteoglycan. Elife 4, e06554 (2015). 10.7554/eLife.06554 PMC4497409 26158506

[b11] Shen G. . Structural basis of the Norrin-Frizzled 4 interaction. Cell Res. 25, 1078–1081 (2015). 26227961 10.1038/cr.2015.92 PMC4559814

[b12] Xu Q. . Vascular development in the retina and inner ear: control by Norrin and Frizzled-4, a high-affinity ligand-receptor pair. Cell 116, 883–895 (2004). 15035989 10.1016/s0092-8674(04)00216-8

[b13] Luhmann U. F. . Role of the Norrie disease pseudoglioma gene in sprouting angiogenesis during development of the retinal vasculature. Invest. Ophthalmol. Vis. Sci. 46, 3372–3382 (2005). 16123442 10.1167/iovs.05-0174

[b14] Ye X. . Norrin, frizzled-4, and Lrp5 signaling in endothelial cells controls a genetic program for retinal vascularization. Cell 139, 285–298 (2009). 19837032 10.1016/j.cell.2009.07.047 PMC2779707

[b15] Xia C. H. . A model for familial exudative vitreoretinopathy caused by LPR5 mutations. Hum. Mol. Genet. 17, 1605–1612 (2008). 18263894 10.1093/hmg/ddn047 PMC2902293

[b16] Gilmour D. F. Familial exudative vitreoretinopathy and related retinopathies. Eye 29, 1–14 (2015). 25323851 10.1038/eye.2014.70 PMC4289842

[b17] MacDonald B. T. & He X. Frizzled and LRP5/6 receptors for Wnt/beta-catenin signaling. Cold Spring Harb. Perspect. Biol. 4, a007880 (2012). 23209147 10.1101/cshperspect.a007880 PMC3504444

[b18] Bilic J. . Wnt induces LRP6 signalosomes and promotes dishevelled-dependent LRP6 phosphorylation. Science 316, 1619–1622 (2007). 17569865 10.1126/science.1137065

[b19] Pan W. . Wnt3a-mediated formation of phosphatidylinositol 4,5-bisphosphate regulates LRP6 phosphorylation. Science 321, 1350–1353 (2008). 18772438 10.1126/science.1160741 PMC2532521

[b20] Yu A., Xing Y., Harrison S. C. & Kirchhausen T. Structural analysis of the interaction between Dishevelled2 and clathrin AP-2 adaptor, a critical step in noncanonical Wnt signaling. Structure 18, 1311–1320 (2010). 20947020 10.1016/j.str.2010.07.010 PMC2992793

[b21] Kim I. . Clathrin and AP2 are required for PtdIns(4,5)P2-mediated formation of LRP6 signalosomes. J. Cell Biol. 200, 419–428 (2013). 23400998 10.1083/jcb.201206096 PMC3575536

[b22] Hagemann A. I. . *In vivo* analysis of formation and endocytosis of the Wnt/beta-catenin signaling complex in zebrafish embryos. J. Cell Sci. 127, 3970–3982 (2014). 25074807 10.1242/jcs.148767 PMC4163645

[b23] Jiang Y., He X. & Howe P. H. Disabled-2 (Dab2) inhibits Wnt/beta-catenin signalling by binding LRP6 and promoting its internalization through clathrin. EMBO J. 31, 2336–2349 (2012). 22491013 10.1038/emboj.2012.83 PMC3364753

[b24] Yamamoto H., Sakane H., Yamamoto H., Michiue T. & Kikuchi A. Wnt3a and Dkk1 regulate distinct internalization pathways of LRP6 to tune the activation of beta-catenin signaling. Dev. Cell 15, 37–48 (2008). 18606139 10.1016/j.devcel.2008.04.015

[b25] Yamamoto H., Komekado H. & Kikuchi A. Caveolin is necessary for Wnt-3a-dependent internalization of LRP6 and accumulation of beta-catenin. Dev. Cell 11, 213–223 (2006). 16890161 10.1016/j.devcel.2006.07.003

[b26] Niehrs C. The complex world of WNT receptor signalling. Nat. Rev. Mol. Cell. Biol. 13, 767–779 (2012). 23151663 10.1038/nrm3470

[b27] Feng Q. & Gao N. Keeping Wnt signalosome in check by vesicular traffic. J. Cell. Physiol. 230, 1170–1180 (2015). 25336320 10.1002/jcp.24853 PMC4433473

[b28] Dobrowolski R. & De Robertis E. M. Endocytic control of growth factor signalling: multivesicular bodies as signalling organelles. Nat. Rev. Mol. Cell. Biol. 13, 53–60 (2012). 10.1038/nrm3244 PMC3374592 22108513

[b29] Vinyoles M. . Multivesicular GSK3 sequestration upon Wnt signaling is controlled by p120-catenin/cadherin interaction with LRP5/6. Mol. Cell 53, 444–457 (2014). 24412065 10.1016/j.molcel.2013.12.010

[b30] Taelman V. F. . Wnt signaling requires sequestration of glycogen synthase kinase 3 inside multivesicular endosomes. Cell 143, 1136–1148 (2010). 21183076 10.1016/j.cell.2010.11.034 PMC3022472

[b31] Metcalfe C. & Bienz M. Inhibition of GSK3 by Wnt signalling--two contrasting models. J. Cell Sci. 124, 3537–3544 (2011). 22083140 10.1242/jcs.091991

[b32] Cruciat C. M. . Requirement of prorenin receptor and vacuolar H+-ATPase-mediated acidification for Wnt signaling. Science 327, 459–463 (2010). 20093472 10.1126/science.1179802

[b33] Mukai A. . Balanced ubiquitylation and deubiquitylation of Frizzled regulate cellular responsiveness to Wg/Wnt. EMBO J. 29, 2114–2125 (2010). 20495530 10.1038/emboj.2010.100 PMC2905240

[b34] de Lau W., Peng W. C., Gros P. & Clevers H. The R-spondin/Lgr5/Rnf43 module: regulator of Wnt signal strength. Genes Dev. 28, 305–316 (2014). 24532711 10.1101/gad.235473.113 PMC3937510

[b35] Hao H. X. . ZNRF3 promotes Wnt receptor turnover in an R-spondin-sensitive manner. Nature 485, 195–200 (2012). 22575959 10.1038/nature11019

[b36] Hao H. X., Jiang X. & Cong F. Control of Wnt receptor turnover by R-spondin-ZNRF3/RNF43 signaling module and its dysregulation in cancer. Cancers 8, 54 (2016). 10.3390/cancers8060054 PMC4931619 27338477

[b37] Koo B. K. . Tumour suppressor RNF43 is a stem-cell E3 ligase that induces endocytosis of Wnt receptors. Nature 488, 665–669 (2012). 22895187 10.1038/nature11308

[b38] Yu A. . Association of dishevelled with the clathrin AP-2 adaptor is required for Frizzled endocytosis and planar cell polarity signaling. Dev. Cell 12, 129–141 (2007). 17199046 10.1016/j.devcel.2006.10.015 PMC2831292

[b39] Nikopoulos K. . Overview of the mutation spectrum in familial exudative vitreoretinopathy and Norrie disease with identification of 21 novel variants in FZD4, LRP5, and NDP. Hum. Mutat. 31, 656–666 (2010). 20340138 10.1002/humu.21250

[b40] Chen W. . Dishevelled 2 recruits beta-arrestin 2 to mediate Wnt5A-stimulated endocytosis of Frizzled 4. Science 301, 1391–1394 (2003). 12958364 10.1126/science.1082808

[b41] Tauriello D. V. . Wnt/beta-catenin signaling requires interaction of the Dishevelled DEP domain and C terminus with a discontinuous motif in Frizzled. Proc. Natl Acad. Sci. USA 109, E812–E820 (2012). 22411803 10.1073/pnas.1114802109 PMC3325702

[b42] Umbhauer M. . The C-terminal cytoplasmic Lys-thr-X-X-X-Trp motif in frizzled receptors mediates Wnt/beta-catenin signalling. EMBO J. 19, 4944–4954 (2000). 10990458 10.1093/emboj/19.18.4944 PMC314225

[b43] Boonstra F. N. . Clinical and molecular evaluation of probands and family members with familial exudative vitreoretinopathy. Invest. Ophthalmol. Vis. Sci. 50, 4379–4385 (2009). 19324841 10.1167/iovs.08-3320

[b44] Veeman M. T., Slusarski D. C., Kaykas A., Louie S. H. & Moon R. T. Zebrafish prickle, a modulator of noncanonical Wnt/Fz signaling, regulates gastrulation movements. Curr. Biol. 13, 680–685 (2003). 12699626 10.1016/s0960-9822(03)00240-9

[b45] Schmidt O. & Teis D. The ESCRT machinery. Curr. Biol. 22, R116–R120 (2012). 22361144 10.1016/j.cub.2012.01.028 PMC3314914

[b46] Bishop N. & Woodman P. ATPase-defective mammalian VPS4 localizes to aberrant endosomes and impairs cholesterol trafficking. Mol. Biol. Cell. 11, 227–239 (2000). 10637304 10.1091/mbc.11.1.227 PMC14770

[b47] Wang Y. . Ephrin-B2 controls VEGF-induced angiogenesis and lymphangiogenesis. Nature 465, 483–486 (2010). 20445537 10.1038/nature09002

[b48] Langton P. F., Kakugawa S. & Vincent J. P. Making, exporting, and modulating Wnts. Trends Cell. Biol. 26, 756–765 (2016). 27325141 10.1016/j.tcb.2016.05.011

[b49] Deng C. . Multi-functional norrin is a ligand for the LGR4 receptor. J. Cell Sci. 126, 2060–2068 (2013). 23444378 10.1242/jcs.123471 PMC3666258

[b50] Zhou Y. & Nathans J. Gpr124 controls CNS angiogenesis and blood-brain barrier integrity by promoting ligand-specific canonical wnt signaling. Dev. Cell 31, 248–256 (2014). 25373781 10.1016/j.devcel.2014.08.018 PMC4223636

[b51] Acebron S. P. & Niehrs C. beta-catenin-independent roles of Wnt/LRP6 signaling. Trends Cell. Biol. 26, 956–967 (2016). 27568239 10.1016/j.tcb.2016.07.009

[b52] Pitulescu M. E. & Adams R. H. Regulation of signaling interactions and receptor endocytosis in growing blood vessels. Cell Adhes. Migr. 8, 366–377 (2014). 10.4161/19336918.2014.970010 PMC4594521 25482636

[b53] Montesano R. . Increased proteolytic activity is responsible for the aberrant morphogenetic behavior of endothelial cells expressing the middle T oncogene. Cell 62, 435–445 (1990). 2379237 10.1016/0092-8674(90)90009-4

[b54] Johnson V., Xiang M., Chen Z. & Junge H. J. Neurite mistargeting and inverse order of intraretinal vascular plexus formation precede subretinal vascularization in Vldlr mutant mice. PLoS ONE 10, e0132013 (2015). 26177550 10.1371/journal.pone.0132013 PMC4503745

