# Supplementary material for: Norrin-induced Frizzled4 endocytosis and endo-lysosomal trafficking control retinal angiogenesis and barrier function
Source: Nat Commun. 2017 Jul 4;8:16050. doi: 10.1038/ncomms16050 (PMC5500887; doi:10.1038/ncomms16050)

Type of file: PDF
Title of file for HTML: Supplementary Information
Description: Supplementary Figures.

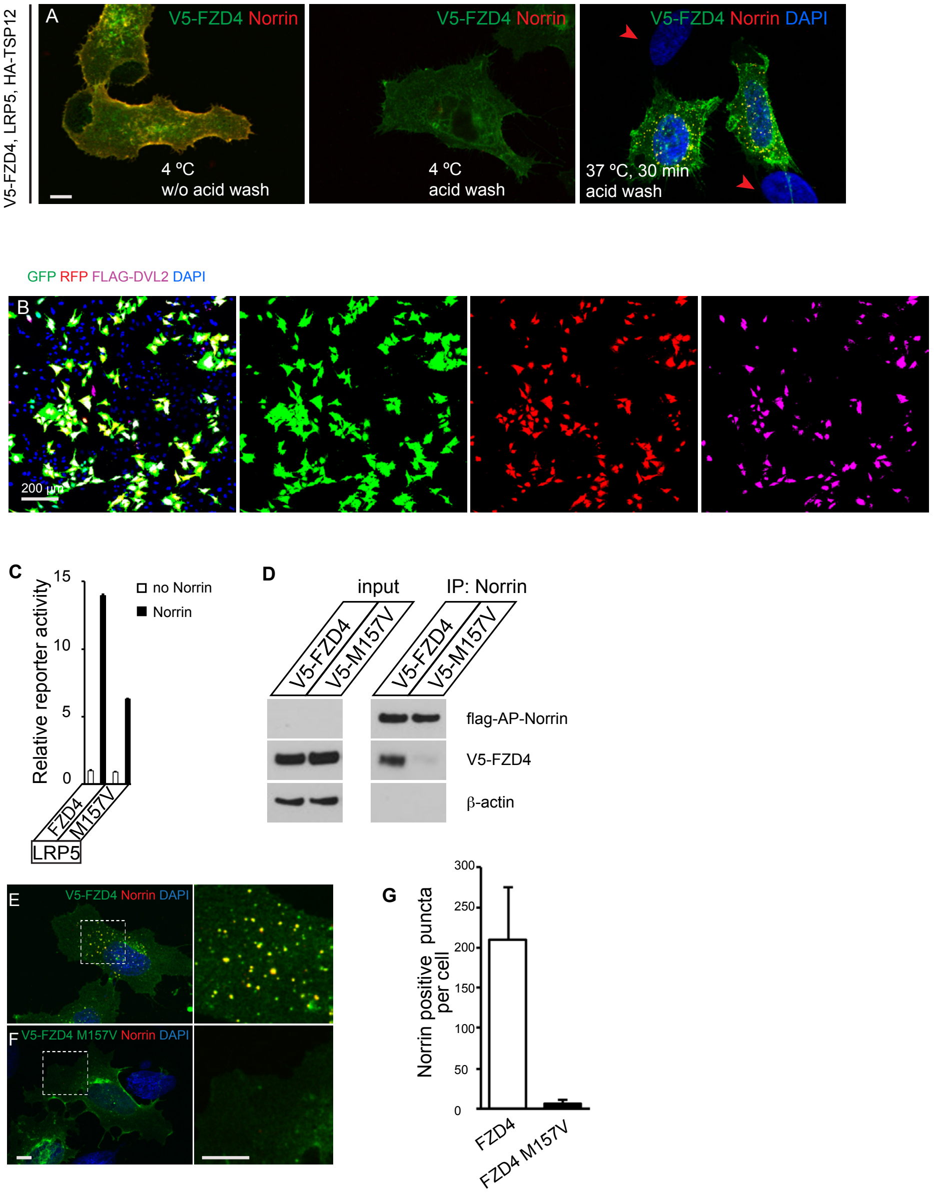

Supplementary Figure 1

Impaired Norrin binding reduces FZD4 endocytosis. **(A)** Norrin binding and internalization in FZD4 expressing cells. A 37 °C incubation step after binding induces endocytosis, the acid wash removes surface bound Norrin. Total V5-FZD4, i.e., internalized and non-internalized FZD4, is revealed by staining for the V5-tag in permeabilized cells. Red arrows highlight untransfected cells, which do not internalize Norrin. (**B**) Indicated constructs were co-transfected into HeLa cells, 3xFLAG-DVL2 was revealed by immunostaining. From 284 GFP positive cells in two fields of view 264 (~93%) were triple positive. (**C**) FZD4 M157V mutation reduces Norrin-induced signaling in TOPFlash assays as expected (Junge et al., 2009). Mean + STDEV shown, n = 3. (**D**) Intact 293T cells expressing V5-FZD4 were incubated with FLAG-AP-Norrin conditioned medium on ice to allow binding to cell surface FZD4. After washing, cells were lysed and Norrin and associated cell surface proteins were isolated using anti-FLAG beads. Levels of cell surface bound Norrin are below the detection limit in the diluted lysate, however, Norrin is efficiently enriched by immunoprecipitation. The M157V mutation reduces Norrin binding. (**E-G**) Norrin-induced internalization of FZD4 is strongly reduced when the interaction of Norrin and FZD4 is disturbed by the M157V mutation. Mean + STDEV shown, n = 20. Scale bars: 10 μm unless otherwise indicated in the figure.

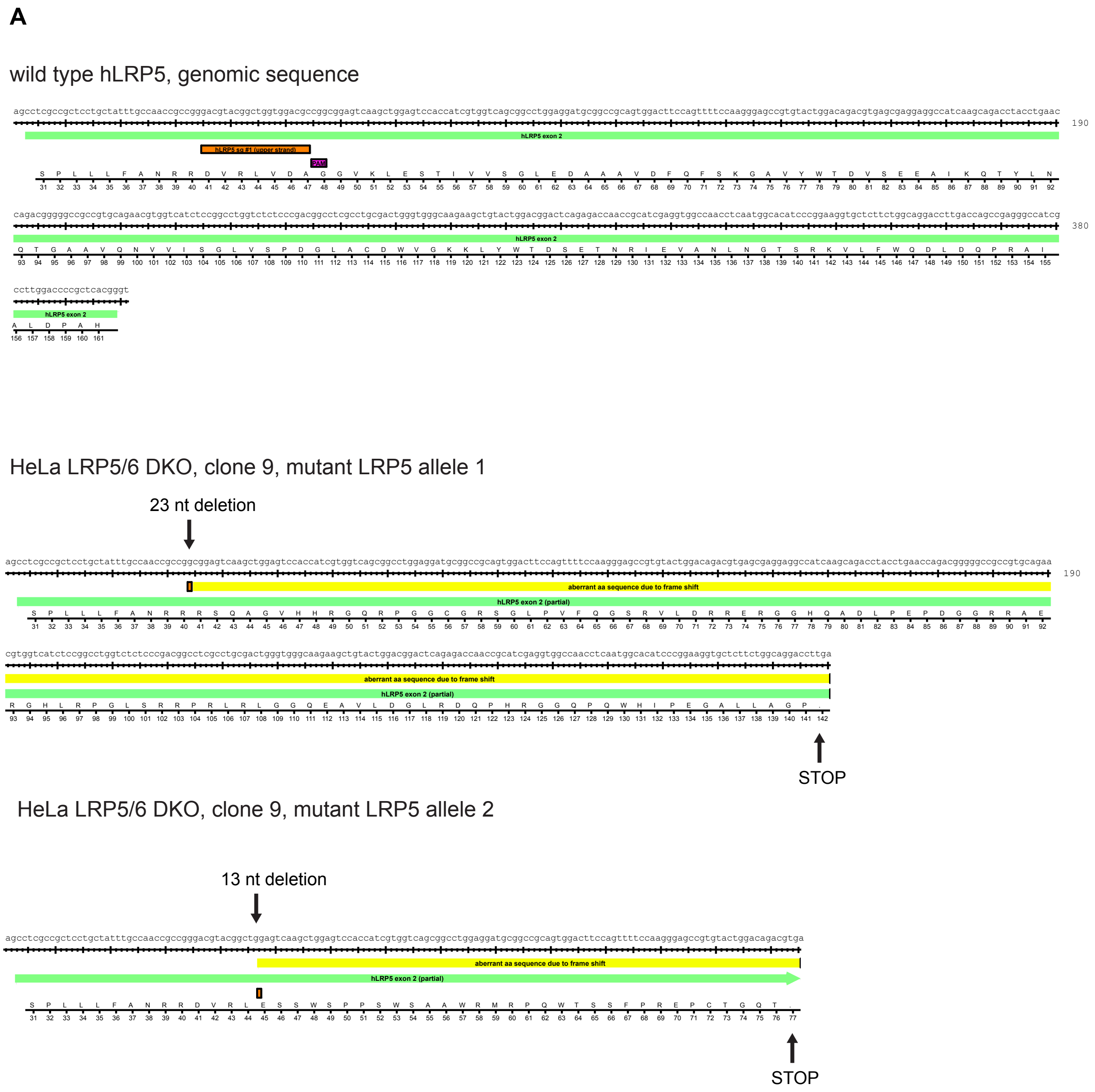

Supplementary Figure 2

CRISPR/Cas9-mediated targeting of LRP5 in HeLa cells. (**A**) LRP5 exon2 and gRNA target sequence are indicated. PAM = protospacer adjacent motif. PCR amplification of the targeted region from a single cell derived HeLa clone, subcloning of the PCR product, and sequencing of 10 amplicons reveals two types of mutated alleles, both of which are null due to frame shifts and early STOPs caused by 23 nt or 13 nt deletions, respectively.

A

wild type hLRP6, genomic sequence

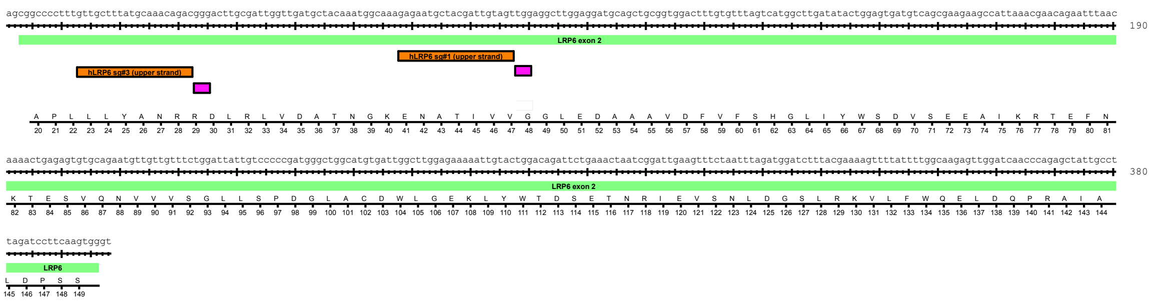

HeLa LRP5/6 DKO, clone 9, mutant LRP6 allele

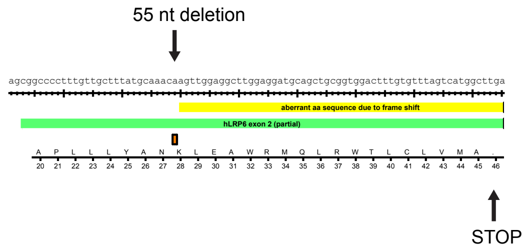

B

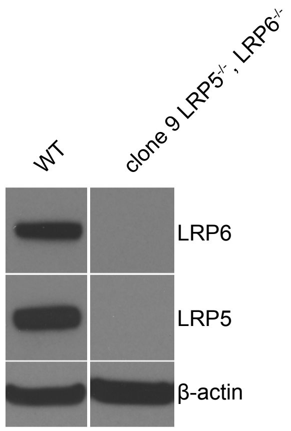

Supplementary Figure 3

CRISPR/Cas9-mediated targeting of LRP6 in HeLa cells. (**A**) LRP6 exon2 and gRNA target sequence are indicated. PCR amplification of the targeted region from a single cell derived HeLa clone, subcloning of the PCR product, and sequencing of 10 amplicons reveals that both LRP6 alleles are targeted in the same manner, such that a 55 nt deletion causes a frame shift and STOP. (**B**) LRP5 and LRP6 cannot be detected in lysates of LRP5/6 double knockout (DKO) cells. The WT and mutant lysates were processed in parallel on the same membrane/film, but interspacing lanes containing lysates from additional clones (not all had the desired DKO genotype) are not shown (see also full scans in Supplementary Fig. 11).

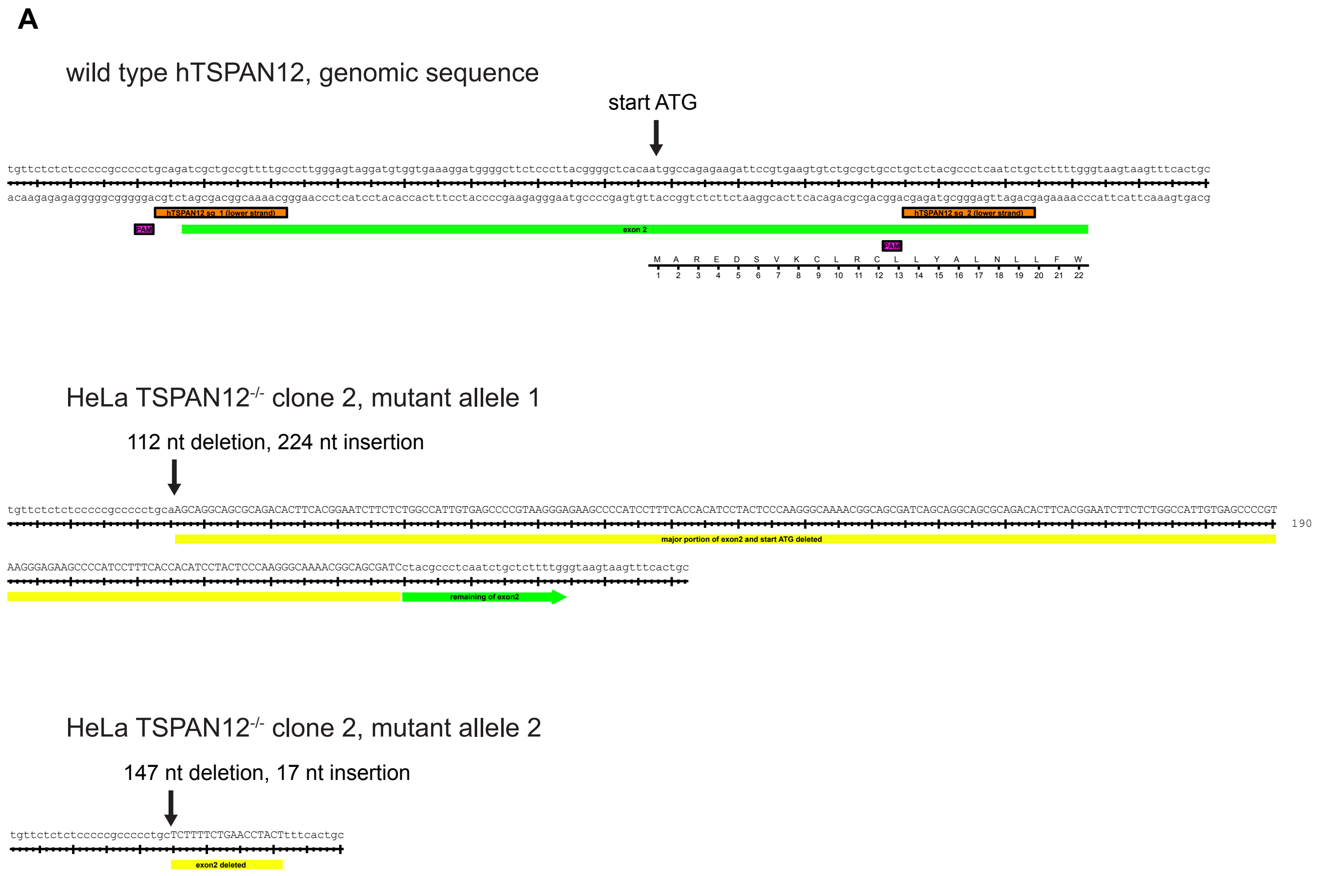

Supplementary Figure 4

CRISPR/Cas9-mediated targeting of TSPAN12 in HeLa cells. (**A**) TSPAN12 exon2 and gRNA target sequence are indicated. PCR amplification of the targeted region from a single cell derived HeLa clone, subcloning of the PCR product, and sequencing of 10 amplicons reveals two types of mutated alleles, both of which are null due to eliminating the start ATG and major portions of the first transmembrane domain. Similar deletions in the mouse genome were used to generate TSPAN12 KO mice (Junge et al., 2009), which show full loss of function in Norrin/FZD4 signaling.

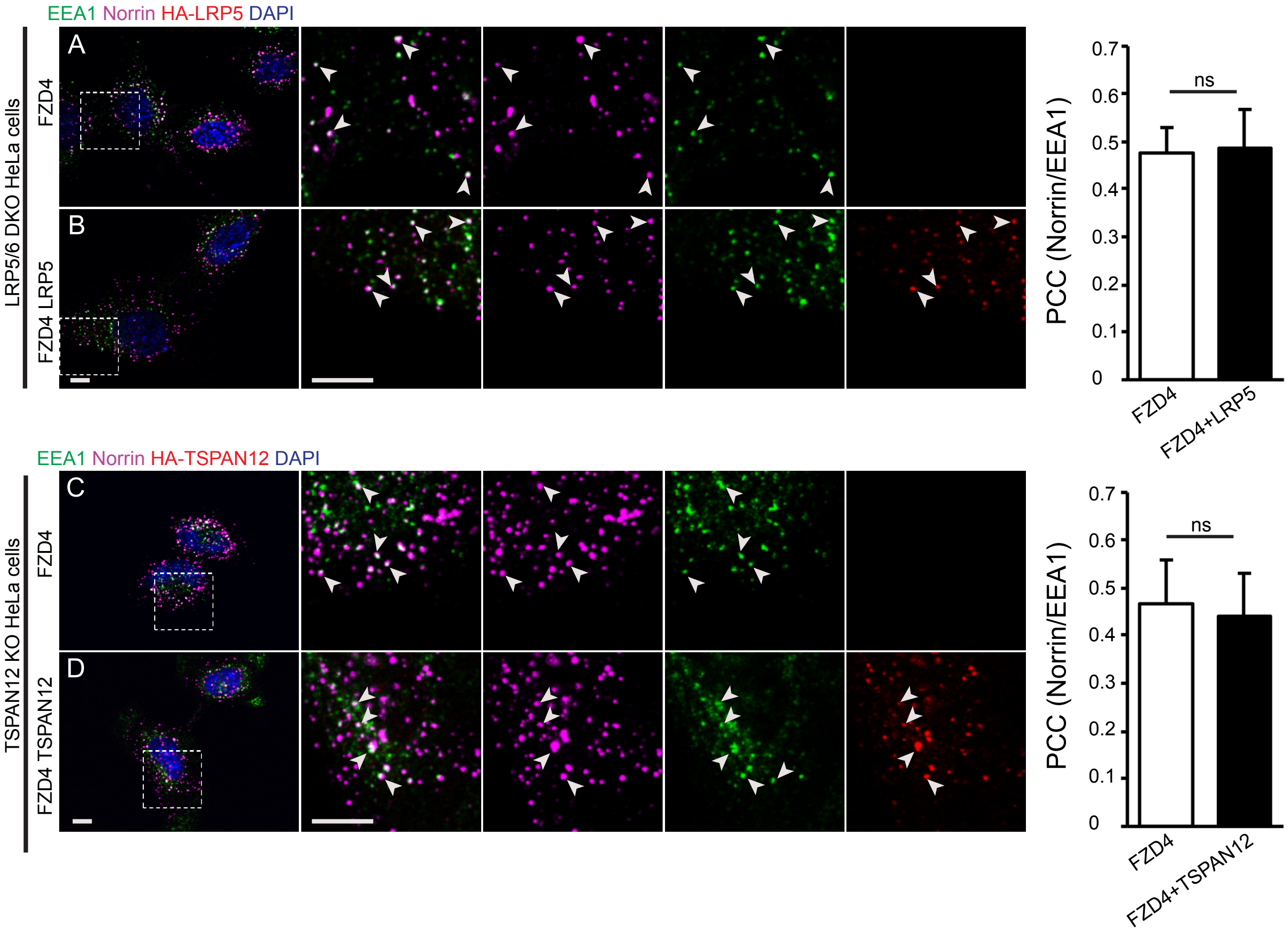

Supplementary Figure 5

Neither LRP5/6 nor TSPAN12 are required for Norrin-induced FZD4 endocytosis. (**A-D**) Norrin efficiently induced FZD4 endocytosis in HeLa cells devoid of LRP5 and LRP6, or, cells devoid of TSPAN12. Restoring LRP5 or TSPAN12 expression did not noticeably alter internalization in cells expressing wild type FZD4. Co-localization of Norrin with EEA1 was quantified using the Pearson correlation coefficient (PCC) (mean + STDEV shown, n=20). Scale bars: 10 µm.

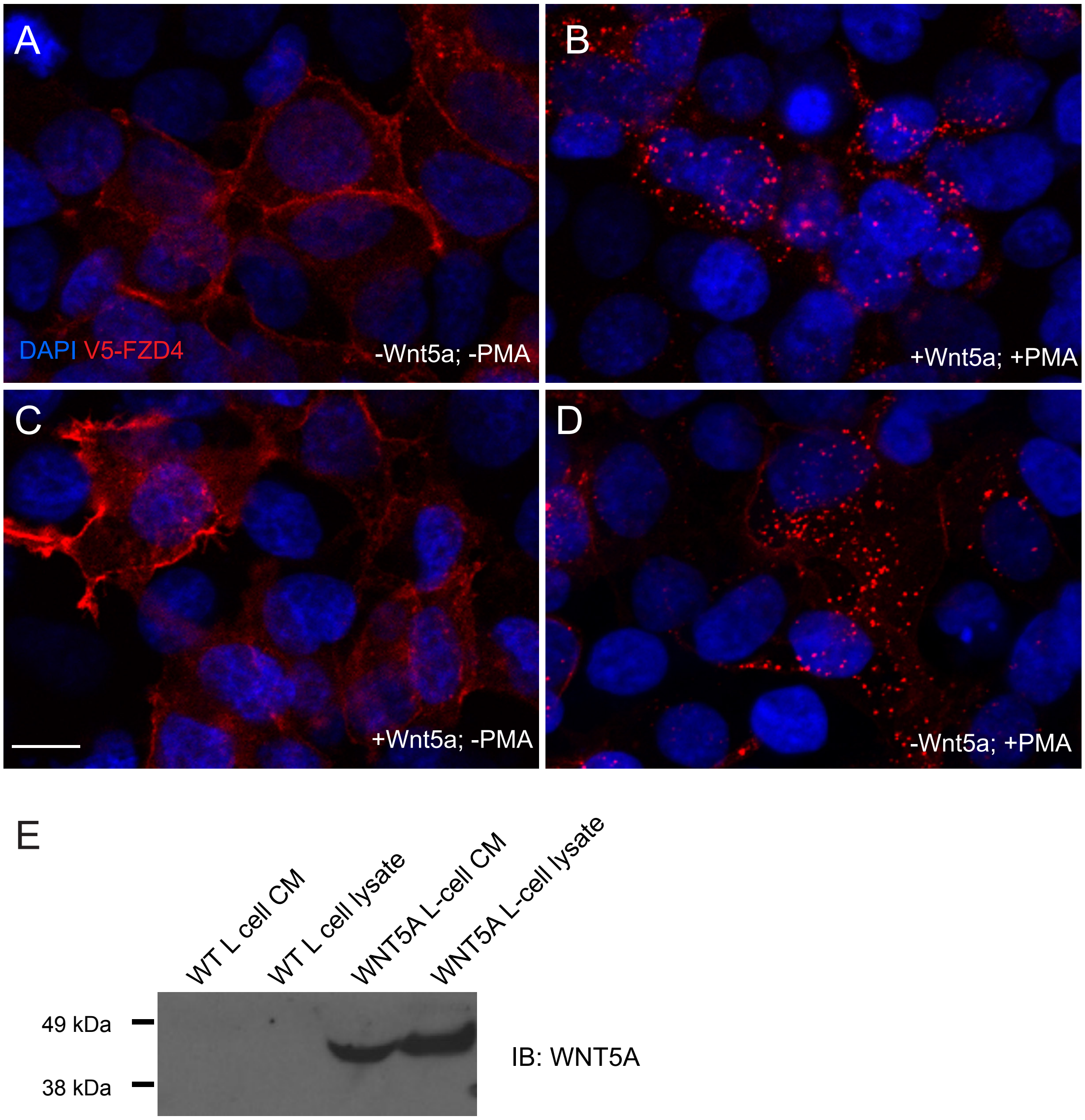

Supplementary Figure 6

WNT5A/PMA-induced FZD4 endocytosis in 293T cells (**A-D**) FZD4 endocytosis could not be induced by WNT5A conditioned medium alone, but 1 μM of the phorbolester PMA is sufficient to induce endocytosis within 1 hr at 37 °C (endogenous WNT5A may be present). (**E**) Robust expression of WNT5A in conditioned medium from L-cells stably expressing WNT5A (ATCC CRL-2814). Scale bar: 10 μm.

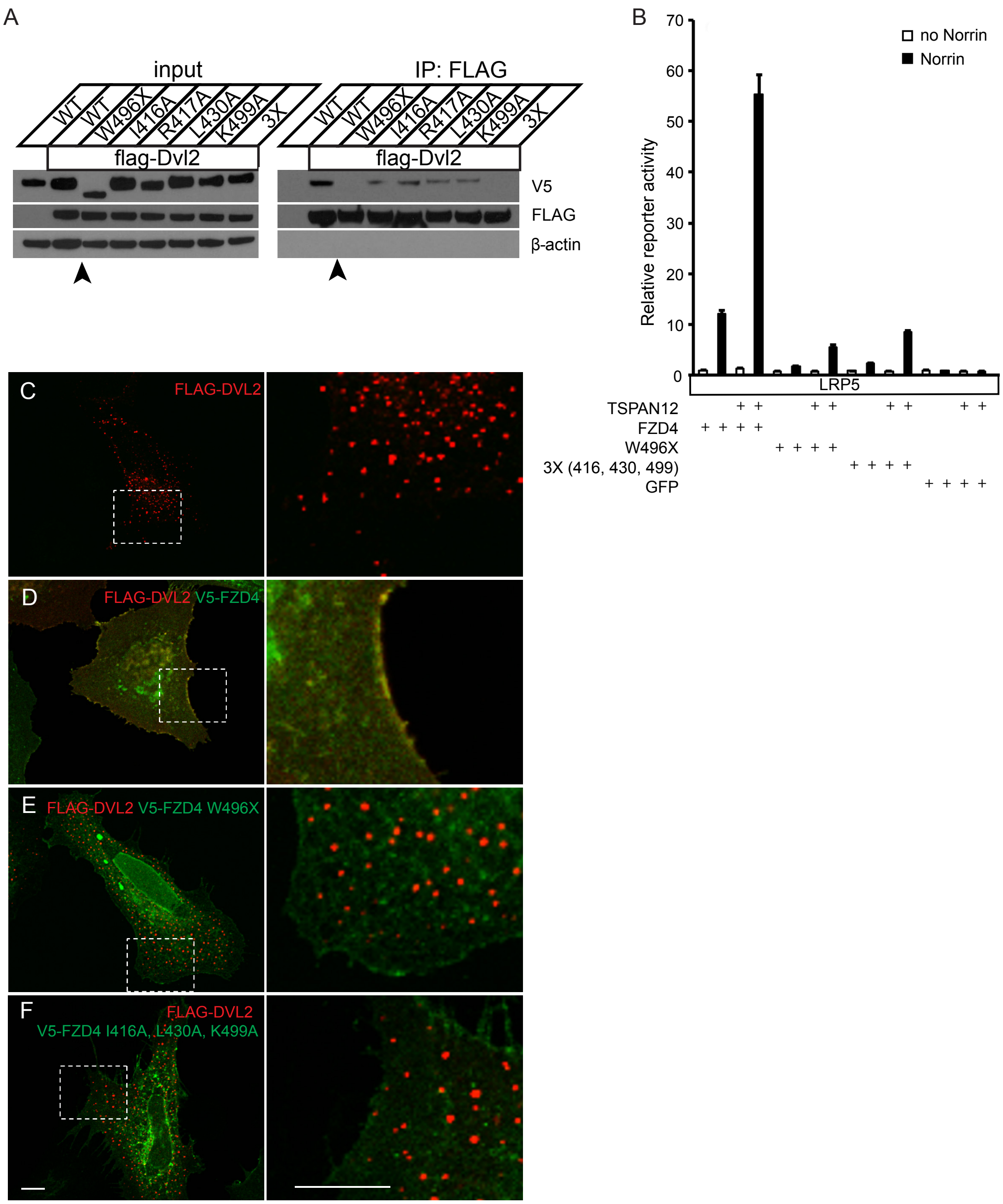

Supplementary Figure 7

Mutations in the intracellular loops of FZD4 abolish DVL2 binding. (**A**) Immunoprecipitation of FLAG-DVL2 expressed in 293T cells co-precipitates V5-FZD4. Point mutations in FZD4 impair DVL2 binding. The W496X truncation present in familial exudative vitreoretinopathy patients and a combination of the mutations I416A, R417A, K499A ("3X") both appear to abolish DVL2 binding within the detection limits of the assay. Arrowheads indicate where a lane has been removed from the image using Photoshop (this lane contained a duplication of lane 3 due to a gel loading error, see also full scans in Supplementary Fig. 11). (**B**) TOPFlash reporter assay in 293T cells transfected with the indicated constructs. Data are normalized to the first bar in each graph (n = 3, mean + STDEV shown). (**C**) HeLa cells transfected with FLAG-DVL2 display cytoplasmic DVL2 aggregates. (**D**) DVL2 was efficiently recruited to the plasma membrane by co-transfected FZD4, preventing the formation of cytoplasmic aggregates. (**E, F**) DVL2 aggregates in cells expressing mutated FZD4 variants which are unable to bind DVL2. Scale bars: 10 μm.

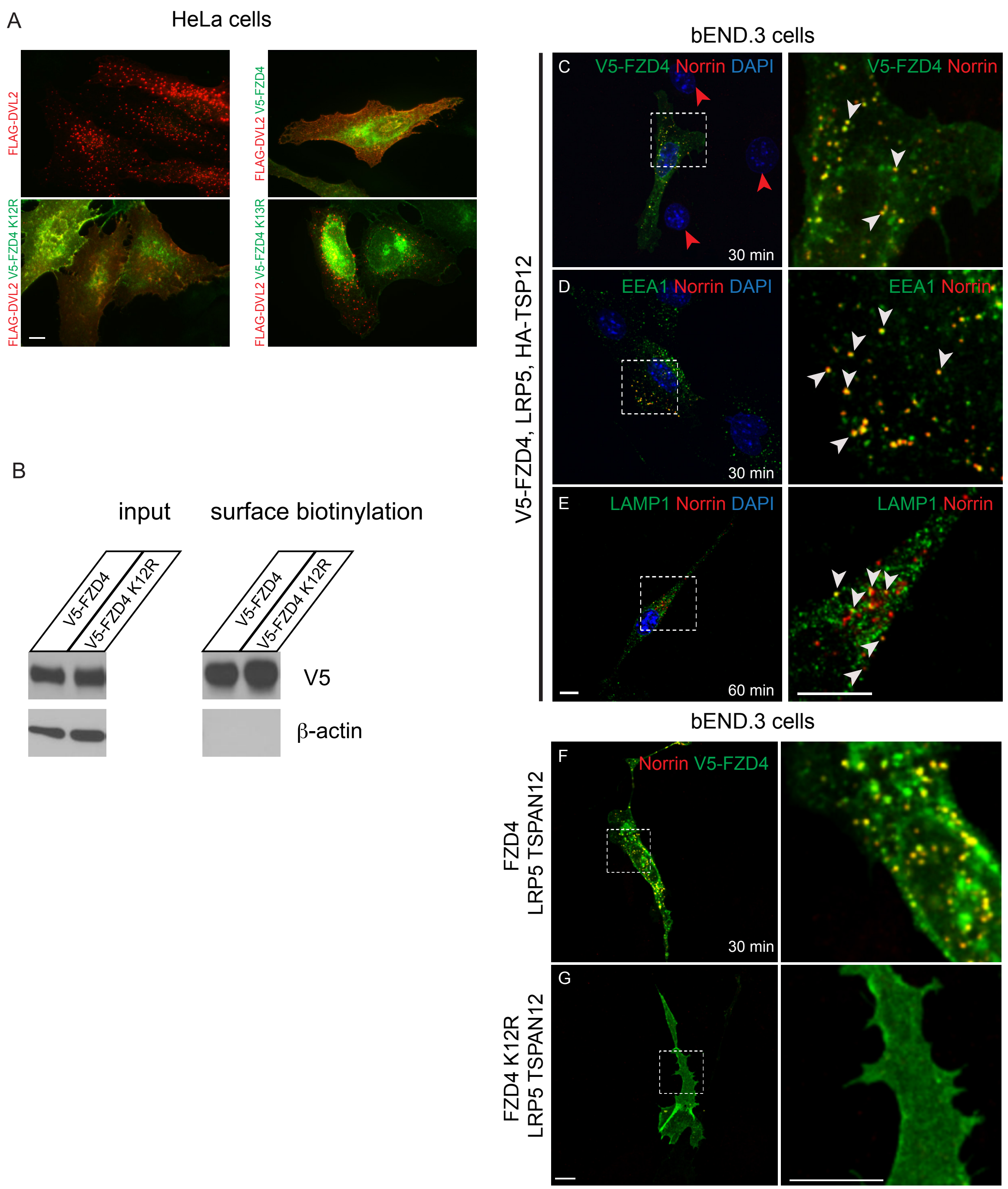

Supplementary Figure 8

FZD4 K12R binds DVL2. (**A**) HeLa cells transfected with FLAG-DVL2 display cytoplasmic DVL2 aggregates. DVL2 was efficiently recruited to the plasma membrane by co-transfected FZD4 or FZD4 K12R, in each case preventing the formation of cytoplasmic aggregates. DVL2 binding to FZD4 K13R was impaired. (**B**) 293T cells were transfected as indicated and plasma membrane proteins of live cells were biotinylated. Cells were lysed and a fraction of the lysate was loaded (input). Biotinylated proteins were isolated with Neutravidin beads and probed with anti-V5 antibody (surface biotinylation). FZD4 K12R levels at the plasma membrane were moderately increased. (**C-E**) Internalized Norrin co-localized with FZD4, EEA1 and LAMP1 in a transformed endothelial cell line, bEND.3. Red arrowheads in C mark untransfected bEND.3 cells, which do not internalize Norrin. (**F-G**). FZD4 K12R, which cannot be ubiquitinated, fails to internalize in response to Norrin stimulation in bEND.3 cells. Scale bars: 10 μm.

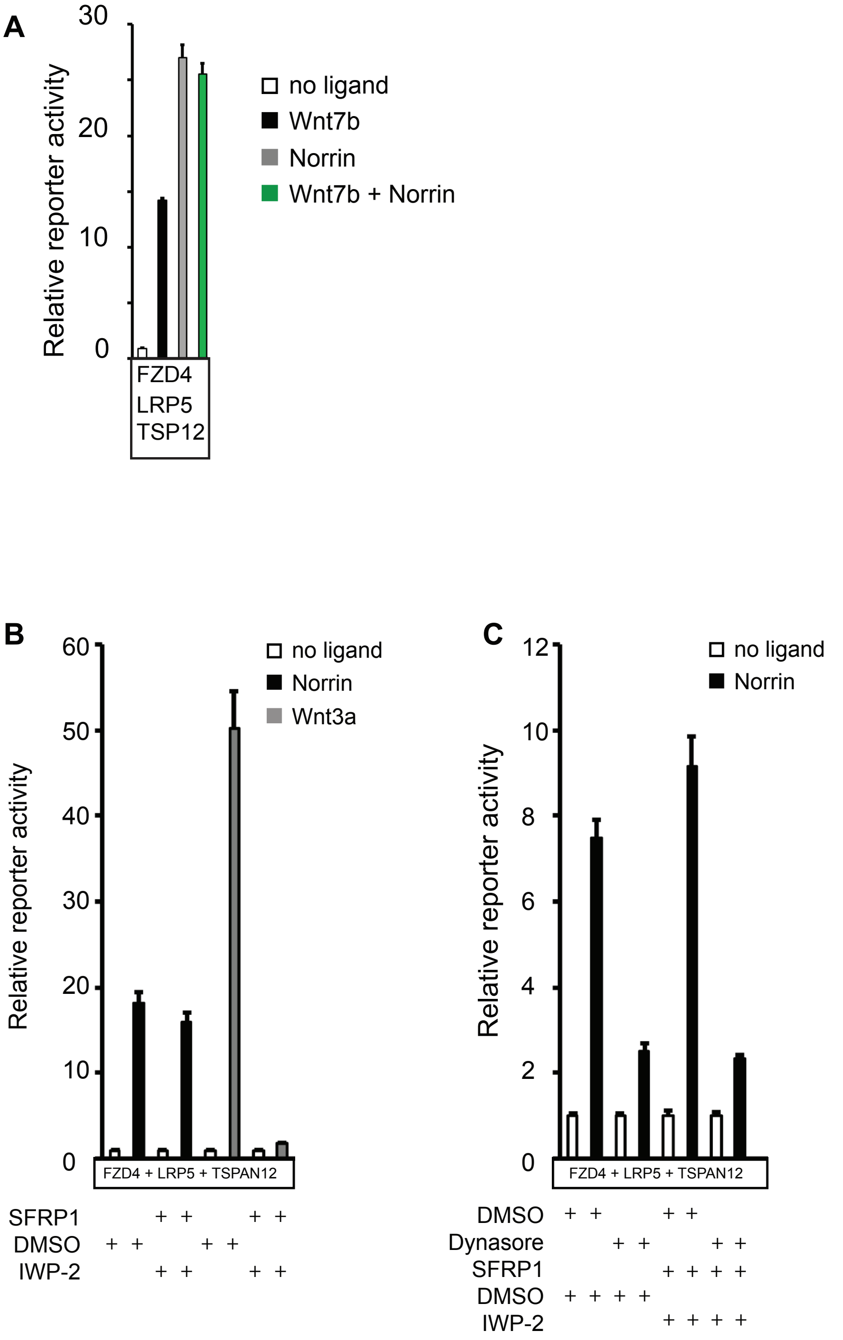

Supplementary Figure 9

Norrin signals independently of endogenous WNTs. (**A**) 293T cells were transfected with indicated constructs. Signaling was induced by co-transfection of Norrin and/or WNT7B expression constructs. Norrin and WNT7B signaling are not additive, likely because Norrin and WNT binding to “site 2” in the extracellular domain of FZD4 is mutually exclusive. (**B**) The combination of sFRP1 co-transfection and IWP-2 effectively blocks signaling induced by co-transfection of WNT3A, whereas Norrin-induced signaling is virtually not affected. (**C**) Dynasore inhibits NDP-induced signaling independent of the activity of endogenous WNTs. A-C, mean + STDEV, n = 3. IWP-2 addition affected the baseline of reporter activity (white bars). To account for that, data in B and C data were normalized to the corresponding data points without ligand stimulation.

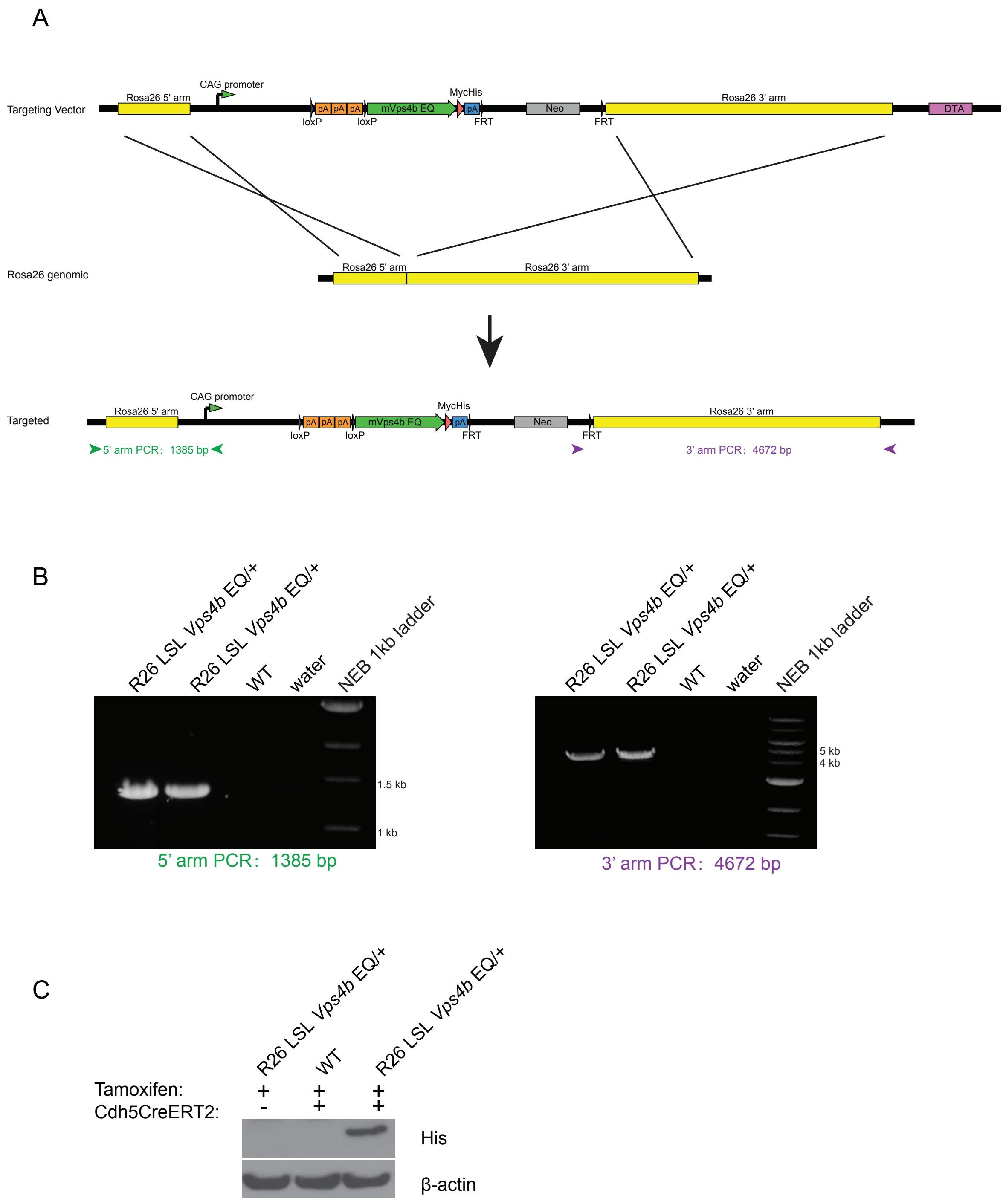

Supplementary Figure 10

Targeting strategy for the generation of *Rosa*26 lox-STOP-lox *Vps4b* EQ mice. (**A**) Transcriptional termination sites (pA) flanked by loxP sites prevent the expression of *Vps4b* EQ with c-terminal mycHis tag unless polyadenylation signals are excised by Cre. CAG promoter: synthetic promoter constructed from several sequences including a CMV enhancer and chicken beta-actin promoter. FRT = flippase recombination site. Neo = neomycin resistance. DTA = Diphtheria toxin A. (**B**) Long-range PCRs confirm the gene targeting event in two *Rosa*26 LSL-VPS4b EQ mice derived from two separate ES cell clones. Identical PCR products were obtained during ES cell screening and confirmed by sequencing. (**C**) *Rosa*26 lox-STOP-lox *VPS4b* EQ expression was induced by tamoxifen injection via the endothelial cell specific VE-Cadherin (Cdh5) CreERT2 driver. Lysates were from lung tissue.

Supplementary Figure 11

In the following full scans of all immunoblot exposures, including molecular weight markers.

full scans for figure 5

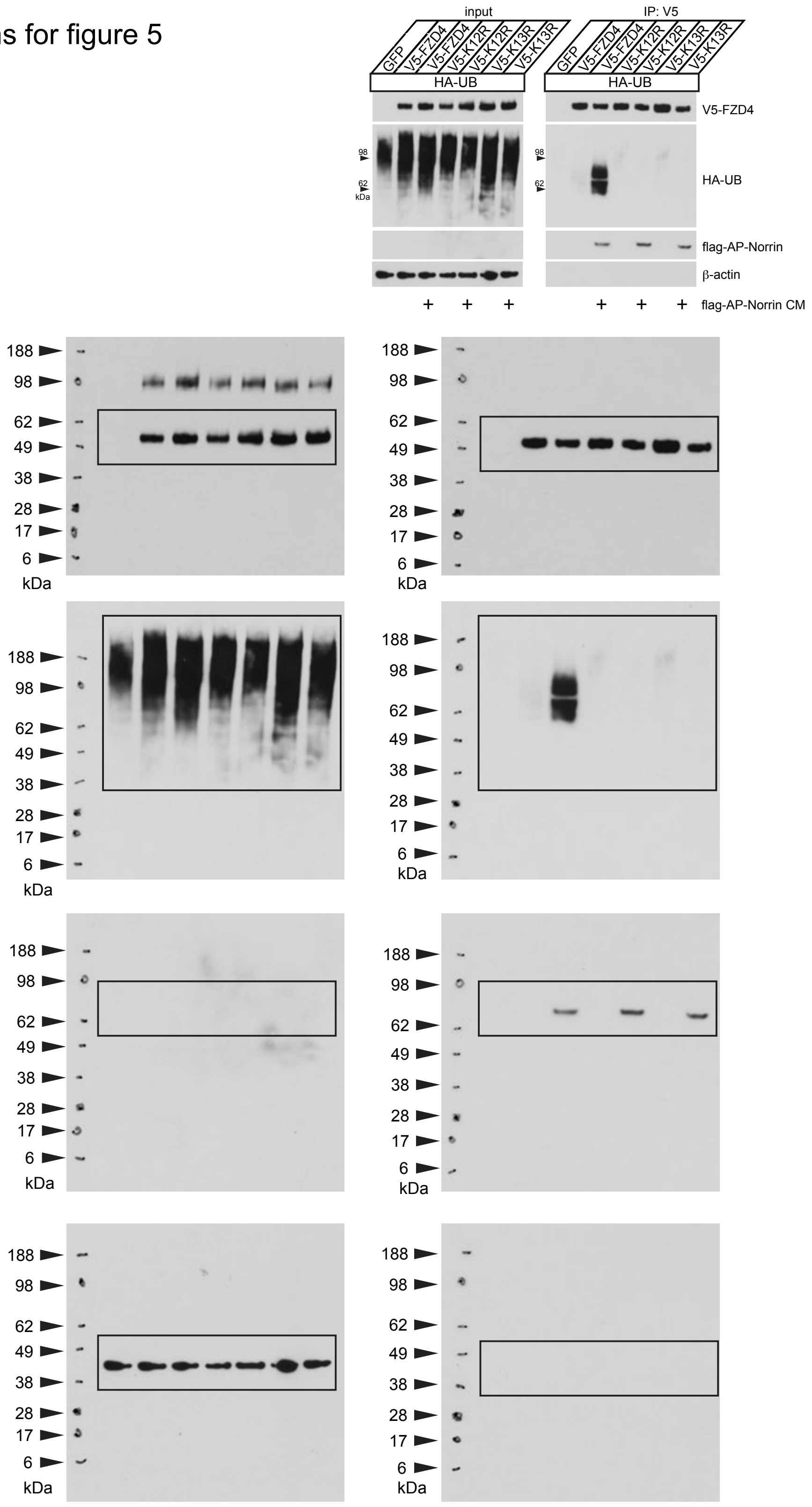

full scans for supplementary figure 1

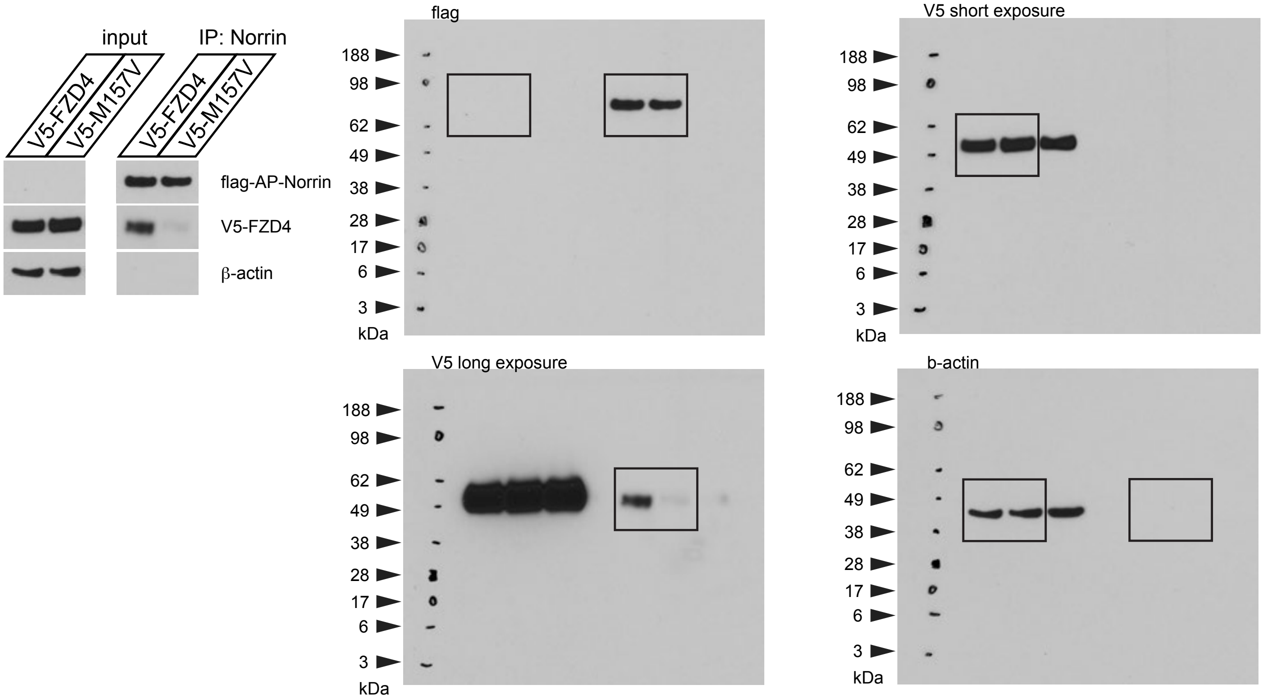

full scans for supplementary figure 3

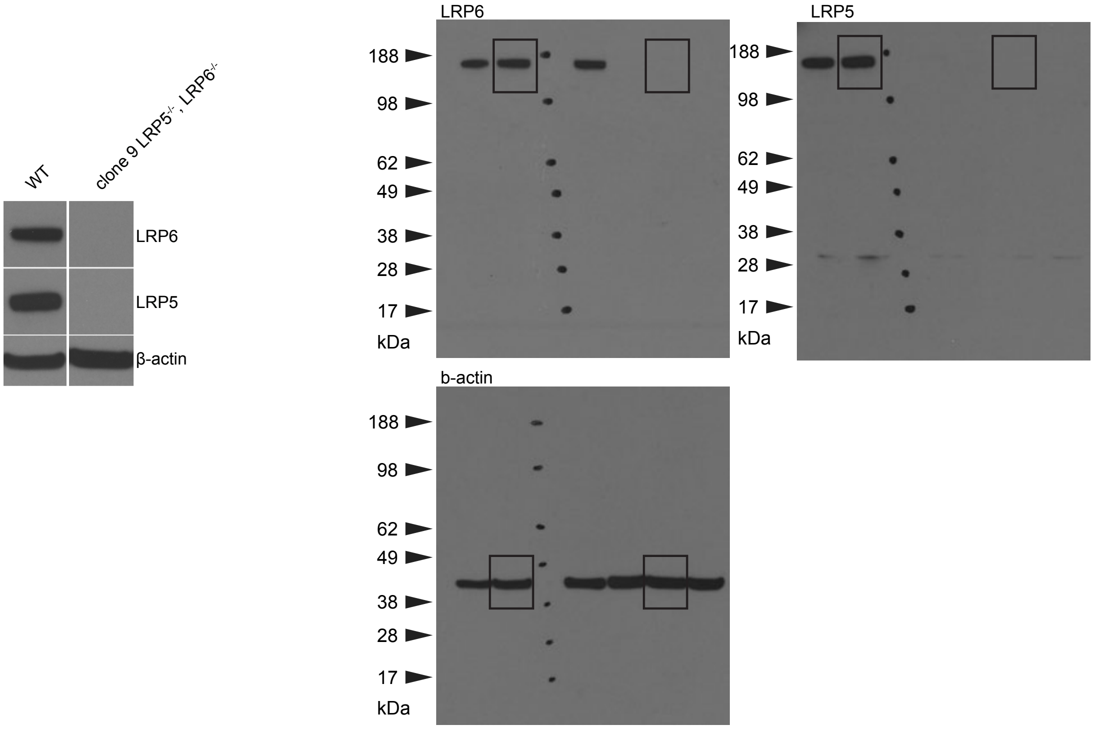

full scan for supplementary figure 6

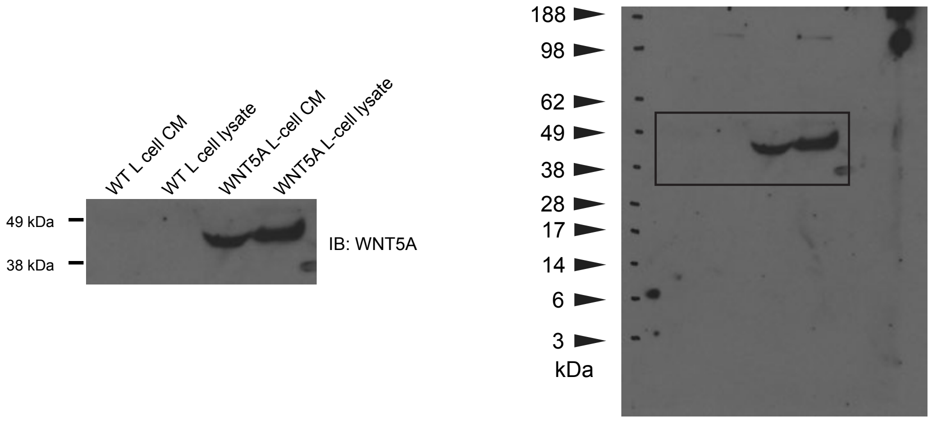

full scans for supplementary figure 7

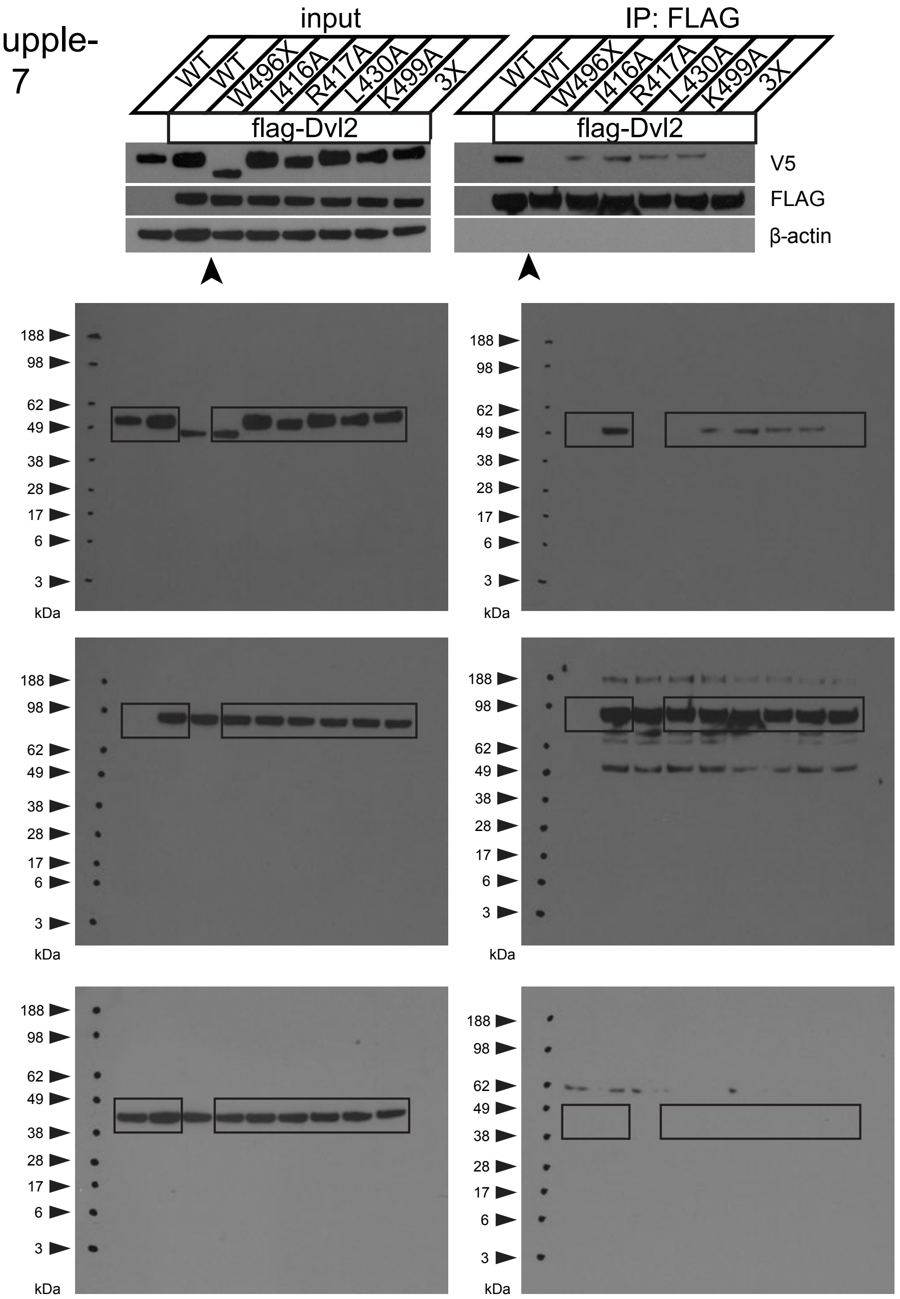

full scans for supplementary figure 8

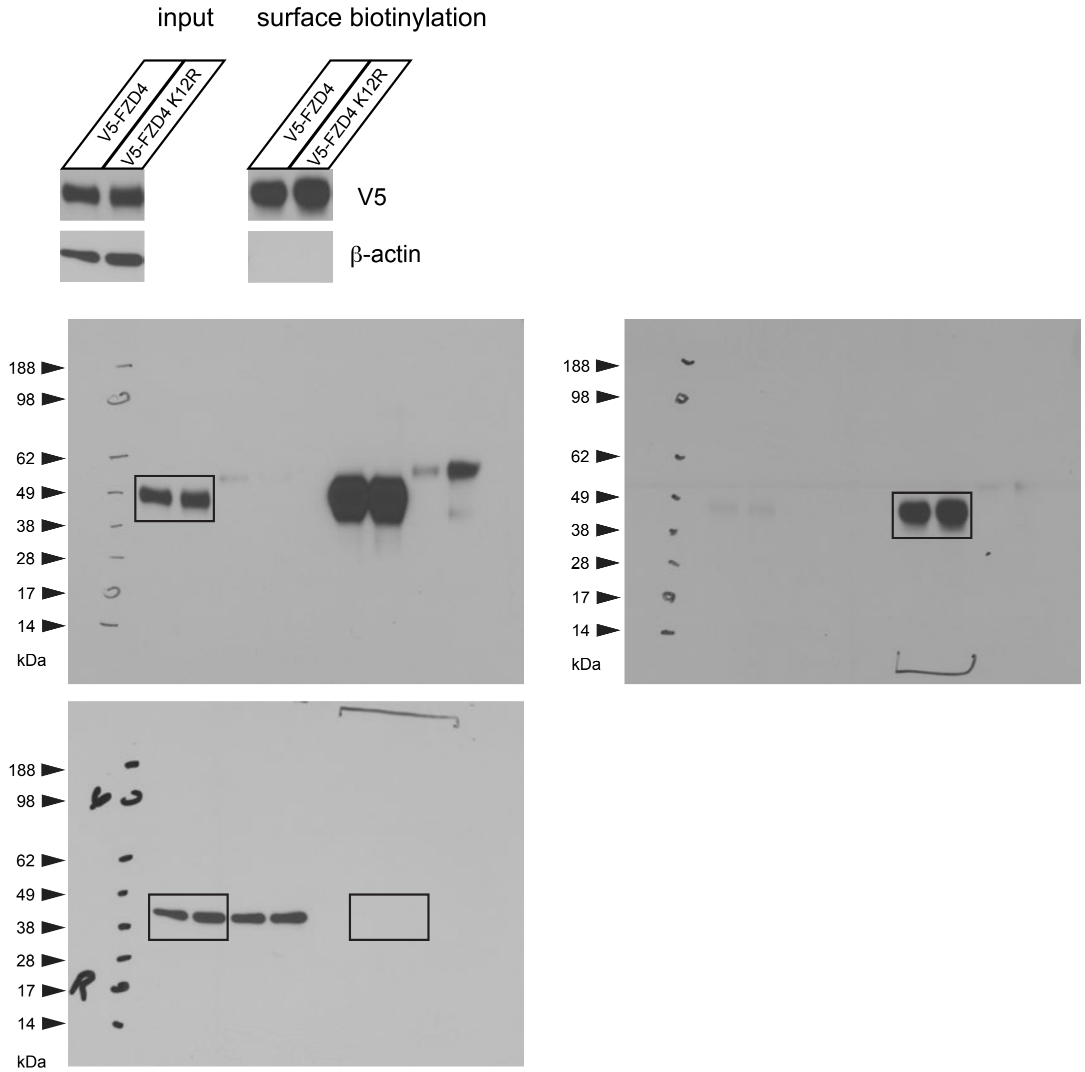

full scans for supplementary figure 10

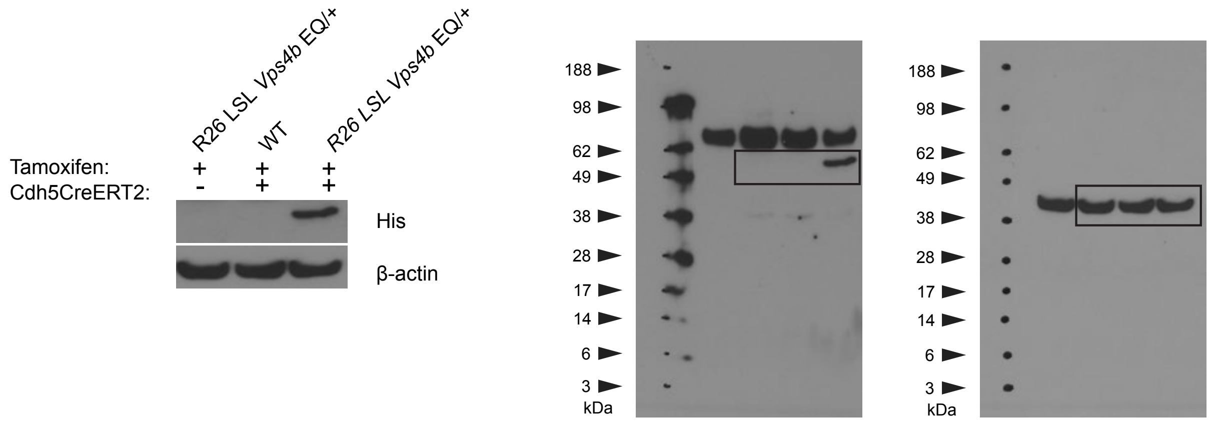

Supplement: Supplementary Information [file ncomms16050-s1.pdf]
